# Immune Status of Individuals with Traumatic Spinal Cord Injury: A Systematic Review and Meta-Analysis

**DOI:** 10.3390/ijms242216385

**Published:** 2023-11-16

**Authors:** Ezra Valido, Gabriela Boehl, Jörg Krebs, Jürgen Pannek, Stevan Stojic, Atanas G. Atanasov, Marija Glisic, Jivko Stoyanov

**Affiliations:** 1 Swiss Paraplegic Research, 6207 Nottwil, Switzerland; 2 Faculty of Health Sciences and Medicine, University of Lucerne, 6003 Lucerne, Switzerland; 3 Clinical Trial Unit, Swiss Paraplegic Center, 6207 Nottwil, Switzerland; 4 Neuro-Urology, Swiss Paraplegic Center, 6207 Nottwil, Switzerland; 5 Department of Urology, Inselspital, Bern University Hospital, University of Bern, 3012 Bern, Switzerland; 6 Ludwig Boltzman Institute for Digital Health and Patient Safety, Medical University of Vienna, 1090 Vienna, Austria; 7 Institute of Genetics and Animal Biotechnology of the Polish Academy of Sciences, 05-552 Magdalenka, Poland; 8 Institute of Social and Preventive Medicine (ISPM), University of Bern, 3012 Bern, Switzerland

**Keywords:** traumatic spinal cord injury, immunology, meta-analysis, immune depression

## Abstract

Individuals with spinal cord injury (SCI) have higher infection rates compared to those without SCI. In this review, the immune status difference between individuals with and without traumatic SCI is investigated by examining their peripheral immune cells and markers. PubMed, Cochrane, EMBASE, and Ovid MEDLINE were searched without language or date restrictions. Studies reporting peripheral immune markers’ concentration and changes in functional capabilities of immune cells that compared individuals with and without SCI were included. Studies with participants with active infection, immune disease, and central nervous system (CNS) immune markers were excluded. The review followed the PRISMA guidelines. Effect estimates were measured by Weighted Mean Difference (WMD) using a random-effects model. Study quality was assessed using the National Heart, Lung, and Blood Institute Quality Assessment Tool. Fifty-four studies (1813 with SCI and 1378 without SCI) contributed to the meta-analysis. Leukocytes (*n* = 23, WMD 0.78, 95% CI 0.17; 1.38, I^2^ 83%), neutrophils (*n* = 11, WMD 0.76, 95% CI 0.09; 1.42, I^2^ 89%), C-reactive protein (CRP) (*n* = 12, WMD 2.25, 95% CI 1.14; 3.56, I^2^ 95%), and IL6 (*n* = 13, WMD 2.33, 95% CI 1.20; 3.49, I^2^ 97%) were higher in individuals with SCI vs. without SCI. Clinical factors (phase of injury, completeness of injury, sympathetic innervation impairment, age, sex) and study-related factors (sample size, study design, and serum vs. plasma) partially explained heterogeneity. Immune cells exhibited lower functional capability in individuals with SCI vs. those without SCI. Most studies (75.6%) had a moderate risk of bias. The immune status of individuals with SCI differs from those without SCI and is clinically influenced by the phase of injury, completeness of injury, sympathetic innervation impairment, age, and sex. These results provide information that is vital for monitoring and management strategies to effectively improve the immune status of individuals with SCI.

## 1. Introduction

Individuals with spinal cord injury (SCI) have a higher risk of respiratory and urinary tract infections compared to individuals without SCI [1,2]. This increased risk is associated with an injury-induced immune depression syndrome (IDS), which is characterized by a low-grade chronic systemic inflammatory state that is commonly observed in this population [3]. The development of SCI-IDS results from (1) the loss of innervation of neuro-immuno-endocrinologic relevant organ systems such as the spleen, adrenals, lymphatic systems, and bone marrow [1,3,4,5]; (2) an imbalance of autonomic nervous system control [1,4,5]; (3) membrane disruptions in otherwise tight and selectively permeable membranes below the SCI [6,7,8]. While the immune system disruption is systemic, much of the published literature focuses on the central nervous system (CNS) and neurologic recovery, which leaves a significant gap in understanding the peripheral immune system that is more related to the higher infection susceptibility in populations with SCI.

Multiple reviews on the immune state of individuals with SCI have been published [1,3,4,5,6,7,8,9,10,11,12,13,14,15], but there are still inconsistencies in what happens to the peripheral immune system in SCI-IDS, such as the immune cell counts and functions. Moreover, none have attempted a quantitative summary of the evidence in the field. To address these gaps, this systematic review aims to explore the differences in concentration and function of the peripheral immune markers between individuals with and without SCI and to investigate the clinical and injury characteristics that can influence the immunological status of individuals with vs. those without SCI.

## 2. Results

### 2.1. Literature Search and Study Characteristics

**Figure 1:**
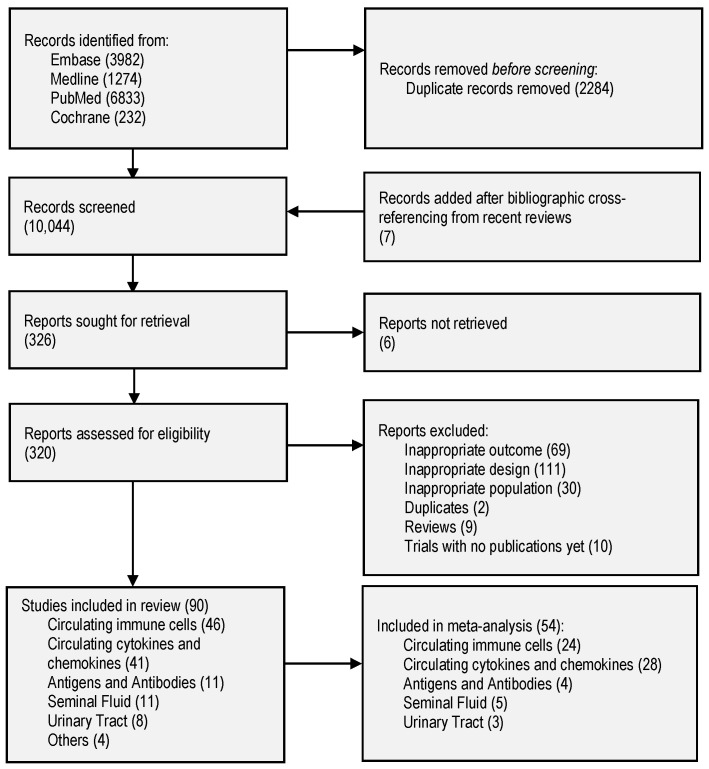
PRISMA flow diagram of included studies.

The initial search identified 12,321 studies from the four databases (Figure 1). After deduplication, 10,037 titles and abstracts were screened, and 312 studies were screened for full text (four studies were not retrieved, and 10 trials were without publications). The four studies were published before 1980 [16,17,18,19]. A total of 90 studies were included Supplemental Table S3). Excluded studies used non-immunological markers, studied animals, or lacked populations with no SCI. A total of 54 (60%) studies were included in the meta-analysis, with 22 immune markers from 3191 study participants (1813 with SCI and 1378 without SCI). The characteristics of this group are summarized in Table 1. The narrative summaries of all included studies can be found in Supplemental Tables S4–S11.

### 2.2. Circulating Immune Markers in SCI

The following immune markers mean concentration were significantly higher in SCI compared to those without SCI: leukocytes (*n* = 23, WMD 0.78, 95% CI 0.17; 1.38, I^2^ 83%), neutrophils (*n* = 11, WMD 0.76, 95% CI 0.09; 1.42, I^2^ 89%), C-reactive protein (CRP) (*n* = 12, WMD 2.25, 95% CI 1.14; 3.56, I^2^ 95%), interleukin (IL)-6 (*n* = 13, WMD 2.33, 95% CI 1.20; 3.47, I^2^ 97%), macrophage migration inhibition factor (MIF) (*n* = 2, WMD 798.51, 95% CI 332.60; 1264.42, I^2^ 7%), chemokine ligand (CXCL)-1 (*n* = 2, WMD 40.09, 95% CI 11.74; 68.45, I^2^ 0%), CXCL-9 (*n* = 2, WMD 96.88, 95% CI 36.68; 157.09, I^2^ 0%), and immunoglobulin M (IgM) (*n* = 2, WMD 0.65, 95% CI 0.26; 1.04, I^2^ 0%). The WMD, confidence intervals (CI), and heterogeneity (I2) are reported in Table 2. In the stratified analysis based on injury level (paraplegia vs. without SCI and tetraplegia vs. without SCI), individuals with tetraplegia showed significantly higher mean concentrations of CRP (*n* = 5, WMD 14.18, 95% CI 6.65; 21.70, I^2^ 92%) and IL6 (*n* = 6, WMD 0.87, 95% CI 0.05; 1.69, I^2^ 81%). Blood immune markers that were pooled for the stratified analysis are summarized in Table 3.

### 2.3. Immune Markers in the Seminal Fluid and Urothelium

Immune cells in the semen demonstrated significantly higher mean concentration of leukocytes (*n* = 3, WMD 9.73, 95% CI 5.21;14.24, I^2^ 81%) and neutrophils (*n* = 2, WMD 4.35, 95% CI 2.37; 6.33, I^2^ 7%) in males with SCI compared to those without injury. In the urothelium, mean mast cell activity was significantly higher in individuals with SCI (*n* = 3, WMD 10.97, 95% CI 4.08;17.86, I^2^ 94%) compared to those without injury.

### 2.4. Immune Cell Capabilities

A total of 14 (15.6%) studies investigated the functional capability of circulating immune cells based on their ability to mature, to phagocytose a pathogen, to produce oxidative bursts, and their cell activity. Comparing individuals with SCI vs. those without SCI in the acute to subacute phase, there are inconsistencies wherein neutrophils and monocytes have higher oxidative capacities with higher adhesion molecules expression while lymphocytes and natural killer cells have no to significantly lower function in individuals with SCI. In the long term, immune cells (lymphocytes, monocytes, natural killer cells, neutrophils, and dendritic cells) in individuals with SCI have no to significantly lower functional capability compared to those without SCI. The results are summarized in Table 4, and the narrative summaries of the studies are in Supplemental Table S12.

### 2.5. Investigation of Heterogeneity and Sensitivity Analyses

Out of 22 performed pooled analyses, heterogeneity was further investigated in six (27.2%) immune markers (Supplemental Table S13). The following clinical and SCI characteristics were identified as potential sources of high heterogeneity: the phase of injury (leukocytes, neutrophils, lymphocytes, monocytes), completeness of injury (leukocytes, lymphocytes), sympathetic innervation impairment (leukocytes), presence of female participants (leukocytes, lymphocytes, IL6), age groups (leukocytes, neutrophils, IL6). The following study design characteristics were potential sources of heterogeneity: sample size (lymphocytes, IL6), study design (lymphocytes), and serum vs. plasma (IL6). Additionally, when fitting a regression line, the WMD of mean blood monocyte levels between SCI vs. non-SCI population decreased with a higher percentage of male study participants (β −0.0017, 95% CI −0.0034, 0, *p* = 0.0486) (Supplemental Table S14). 

The leave-one-out (LOO) analysis showed that no single study significantly influenced the effect estimate in leukocytes, monocytes, TNFα, IL6, and CRP in individuals with SCI compared to those without SCI (Supplemental Figure S1A–E). When comparing individuals with vs. without SCI, omitting the study of Lin et al. (WMD 0.38, 95%CI −0.09; 0.84, I^2^ 75%) or of Bao et al. [21] (WMD 0.62, 95%CI −0.06; 1.30, I^2^ 89%) or of Pavlicek et al. [34] (WMD 0.75, 95%CI −0.03; 1.52, I^2^ 90%) or of Iversen et al. [35] (WMD 0.82, 95%CI −0.04; 1.69, I^2^ 90%) resulted in a non-significant shift in the estimate for the neutrophil concentration (Supplemental Figure S1F). On the other hand, omitting the study of Iversen et al. [35] (WMD −0.18, 95%CI −0.32; −0.04, I^2^ 68%) resulted in a significant shift in the estimate for the lymphocytes towards lower concentrations among those with SCI compared to those without SCI (Supplemental Figure S1G). When comparing individuals with tetraplegia vs. those without SCI, omitting the study of Iversen et al. [27] (WMD −0.19, 95%CI −0.34;−0.03, I2 60%) resulted in a significant shift in the estimate for the lymphocytes towards lower concentrations among those with tetraplegia compared to those without SCI (Supplemental Figure S2B). 

### 2.6. Quality of Studies and Publication Bias

With the risk of bias assessment, 17 (18.9%) were with low risk, 68 (75.6%) with moderate risk, and 5 (5.6%) with high risk (Supplemental Tables S1 and S2). Most observational studies did not provide sufficient justification for sample size or a power description and did not differentiate levels of exposure or characterization of SCI. All interventional studies were non-randomized controlled studies with participants and assessors not blinded, and there was no report of sample size justification. Furthermore, no significant funnel plot asymmetry was detected for leukocytes, neutrophils, lymphocytes, monocytes, IL6, and TNFα (Supplemental Figures S3 and S4; Supplemental Table S15). However, CRP showed significant funnel plot asymmetry (Egger intercept 3.87, *p*-value 0.0039), primarily due to a lack of studies reporting lower CRP concentrations in individuals with SCI compared to those without SCI. No significant funnel plot asymmetry was detected for leukocytes, lymphocytes, and monocytes in the studies with tetraplegia compared to those without SCI (Supplemental Figure S5; Supplemental Table S16).

## 3. Discussion

**Figure 2:**
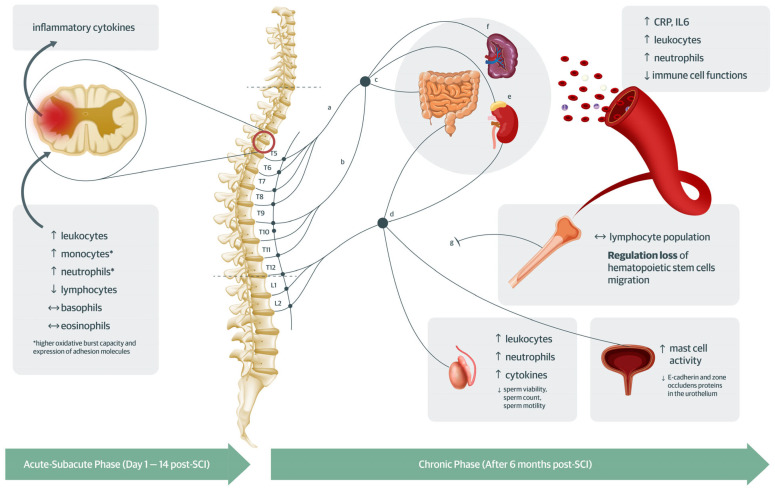
The pathophysiological process involved in SCI-IDS as captured in this systematic review. At the acute–subacute phase, the immune status is mainly driven by the injury with recruitment of leukocytes primarily by neutrophils and monocytes with higher release of inflammatory cytokines that direct the immune cells. Upon resolution of the lesion site inflammation, there is persistence of low-grade inflammation and higher levels of circulating leukocytes, particularly neutrophils. This is secondary to (1) the loss of sympathetic innervation in immune-related organs, (2) exposure of the immune cells to hormone imbalance, particularly to cortisol and norepinephrine, and (3) the membrane disruptions and tissue remodeling after loss of innervation as seen in higher cytokines and immune cells in previously selectively permeable or impermeable membranes (e.g., like in the testis) or increased activity of mucosal immune cells with decreased expression of junction proteins in the urothelium. Legend: a—greater splanchnic nerve; b—lesser splanchnic nerve; c—celiac ganglion; d—inferior mesenteric ganglion; f—spleen; e—adrenal glands; g—sympathetic nerves depending on the bone marrow site; **↓** significantly lower in SCI vs without SCI, **↑** significantly higher in SCI vs without SCI, ↔ no significant difference in SCI vs without SCI.

In this systematic review and meta-analysis, we conducted a comprehensive synthesis of immune markers to enhance the understanding of SCI-IDS (Figure 2). We identified SCI-relevant characteristics (phase of injury, completeness of injury, sympathetic innervation impairment, age, and sex) as potential modifiers in their immune status. The findings show that compared to individuals without SCI, individuals with SCI had (1) significantly higher mean peripheral leukocyte concentration, (2) significantly higher mean peripheral cytokine concentrations, and (3) significantly lower peripheral immune cell functions in the chronic phase. However, the findings are mostly based on cross-sectional studies, which has the potential risk for reverse causality and should be interpreted with caution. 

The phase of injury was seen as a modifier in the immune response of individuals with SCI. In the acute–subacute phase, the leukocyte concentrations are mainly driven by higher neutrophils, higher monocyte levels, and lower lymphocyte levels. This indicates that the injury induces an immune response that is directed to the lesion that may be facilitated by multiple cytokines at the acute–subacute phase [1,4,14,15,36,37,38]. This interplay of pro- and anti-inflammatory cytokines and immune cell migration at the acute–subacute phase is likewise seen in gene expression studies [39,40]. The control of these initial immune-inflammatory processes may help improve the neurologic recovery [5,11,13,41], and recent studies look into the utility of neutrophil to lymphocyte ratio as a predictor for this recovery [14,42,43]. In the chronic phase, CRP was significantly higher in SCI compared to individuals without SCI. Low-grade chronic inflammation characterizes SCI populations, and this is related to secondary consequences of SCI [1,3,6,7]. Membrane permeability disruptions and possible tissue remodeling post-SCI contribute to this chronic inflammation. We found significantly higher immune markers in semen and heightened mast cell activity in the urothelium. This is accompanied by significantly lower observed junction proteins in the urothelium of individuals with SCI vs. those without SCI [44,45,46]. Similarly, in the gut, there is neuromuscular remodeling [47] and bacterial translocation, the transfer of bacteria into the bloodstream from the gut, in animal studies post-SCI [48]. This contributes to higher pathogen-to-immune system crosstalk that leads to higher immune cell mobilization and inflammation, which results in changes in the gut microbiome in populations with SCI as well [6,8,49]. 

Having a complete or incomplete SCI affects the immune status. The subgroup analysis showed that the group with incomplete SCI had significantly higher concentrations of leukocytes and significantly lower concentrations of lymphocytes compared to those without SCI. Individuals with complete SCI have a higher probability of having sympathetic nerve plexus impairment. The sympathetic nervous plexus is at the thoraco-lumbar section of the spine, and it is crucial for hormonal balance, inhibitory bone marrow stimuli, and visceral organ innervation [1,4,6,50]. The loss of sympathetic control leads to an imbalance of hormonal releases, particularly norepinephrine and cortisol, that affects the immune cells’ maturation, leading to lower functional capabilities of these cells [4,25]. A significantly lower immune cell function has been seen in the studies in this review at the chronic phase. The loss of inhibitory bone marrow control likewise results in the unregulated release and migration of immature immune cells [4,27]. Moreover, the level of the injury also plays a role and has been observed in animal models as a modifier of immune response [51,52,53]. Individuals with tetraplegia have disrupted sympathetic innervation at a high level, while those with paraplegia have varying degrees of sympathetic innervation impairment at the thoraco-lumbar level. Results from the stratified analysis showed that individuals with tetraplegia had significantly higher CRP and IL6 concentrations and significantly lower granulocyte concentrations compared to those without SCI, and no significant differences were found in the immune markers tested with those with paraplegia vs. those without SCI. 

Age was identified as a modifier of immune status in the review. The “With Seniors” group had significantly higher leukocytes, neutrophils, and IL6 concentrations but lower lymphocyte concentrations compared to individuals without SCI. The immune system of elderly individuals without SCI is characterized by elevated inflammatory cytokines, normal leukocyte and neutrophil concentrations, decreased lymphocyte concentration, and reduced immune cell functions [54,55,56]. Some studies hypothesized that the immune system following SCI may be aging more rapidly [34]. However, caution must be exercised when interpreting these results in the review as the “Adult” group primarily consisted of physically active males, which potentially confounds the association.

Most SCI studies predominantly involved male participants with a notable lack of female representation, as observed in the literature [57]. This is due to the lower incidence of SCI in females and the exclusion of females from studies due to hormonal differences between the sexes. The presence of females with SCI showed higher concentrations of leukocytes and IL6 but lower lymphocyte concentration vs. individuals without SCI. This indicates that sex is a modifier of the immune status in this population. Sex differences in immune status are documented in the literature, with females without SCI having higher lymphocyte levels compared to males [58]. The immune status in individuals with SCI is affected by hormonal differences between sexes and is likely linked to the disrupted reproductive endocrinologic system below the lesion level [9]. 

### Strengths and Limitations of the Study

This review employed a sensitive search strategy to examine the published literature without restrictions, resulting in the inclusion of many studies. Quantitative summary estimates of 26 immune markers in the blood, semen, and urothelium in clinical studies with analysis of the sources of heterogeneity were performed. The population of SCI in the review had no active infection or immune disease. We acknowledge that some studies were not included in the pooled data due to the unavailability of values despite contacting corresponding authors. Moreover, the medications, particularly at the acute–subacute phases, could have affected the effects on immune cells and markers, and these were not reported by the studies. Key limitations were that most studies were cross-sectional, there was no adjustment for confounders in the analysis, and there was substantial heterogeneity in the pooled estimates.

The existing literature on SCI-IDS, especially regarding the peripheral immune system, is limited. A more thorough characterization of the injury level, completeness, and sympathetic innervation impairment, with age and sex, should be consistently reported. This review focused on routine measurements of immune cells and immune-related cytokines and excluded omics-based studies. Omics-based studies are good starting points to investigate further the modifiers observed in this review. Longitudinal clinical studies are currently limited, and there is a need to investigate interventions that improve the peripheral immune system. Moreover, interventions should also aim to correct the immune-endocrinological imbalance present in this population and not only focus on sensory–motor function recovery. 

## 4. Materials and Methods

### 4.1. Data Sources and Search Strategy

This review was conducted in accordance with the Preferred Reporting Items for Systematic Reviews and Meta-Analysis (PRISMA) statement [59] and the guidelines provided by Muka et al. [60]. The review protocol was registered with the International Prospective Register of Systematic Reviews (PROSPERO No. CRD42021279195). Electronic searches were performed via PubMed, Cochrane CENTRAL, EMBASE, and Ovid MEDLINE from inception until 11 January 2023. The search was conducted by the primary author and developed from previous search strategies that were published by the authors [18,19]. Additional articles were identified through a bibliography screening of reviews published since 2020 [1,7,11,12,14,15,61]. The search strategy can be found in Supplemental Appendix SI. Corresponding authors of eligible studies were contacted via email or ResearchGate for additional data. Studies without online versions were obtained with the assistance of librarians at the Swiss Paraplegic Research Library. 

### 4.2. Study Selection, Eligibility Criteria, and Data Extraction

Both observational and interventional studies were included. Inclusion criteria were studies involving (a) adult individuals with traumatic SCI that are compared to individuals without SCI and (b) studies that measured immune markers. Exclusion criteria were (a) studies using samples derived from the central nervous system, (b) non-traumatic SCI, (c) studies with populations with underlying infectious diseases or immune system disorders, (d) omics studies, (e) animal studies, and (f) reviews, case series, case studies, conference abstracts, letters to editors, commentaries, and other non-peer-reviewed articles. No language or year restrictions were applied. 

Two reviewers independently screened titles and abstracts with selected articles undergoing full-text assessment based on inclusion and exclusion criteria. Differences between reviewers were resolved by a third reviewer. Data extraction was performed independently using pre-designed templates as described in the protocol. 

### 4.3. Risk of Bias and Quality of Evidence Assessment

The quality and risk of bias were independently assessed by two reviewers based on study type. Studies were evaluated using the National Heart, Lung, and Blood Institute Quality Assessment Tool. Details of the assessment tools can be found in Supplemental Tables S1 and S2. 

### 4.4. Data Synthesis and Analysis

Immunological markers’ mean values, median, interquartile range, standard deviation (SD), or standard error of the mean were extracted. Means and SD were computed using established methods for values expressed as medians and ranges [62]. The Weighted Mean Difference (WMD) between SCI and those without SCI was calculated via a random-effects model by DerSimonian and Laird method [63]. Heterogeneity was explored through Cochran’s squared test (χ^2^) and Higgins I^2^ statistic test, as well as subgroup analyses (categorical variables) and meta-regression (continuous variables). SCI-relevant variables (phase of injury [acute_subacute phase vs. chronic], completeness of the injury [complete vs. mixed], sympathetic innervation impairment [above T6 involvement vs. mixed], presence of female participants [all males vs. with females] and age groups [adults vs. with seniors]) and study characteristics (sample size [more than 30 vs. less than 30], geographic location [Asia and the Pacific vs. Europe and the Americas], risk of bias [low vs. moderate_high risks], study design [observational vs. interventional) were used to investigate heterogeneity. The phase of injury is classified as acute (first 48 h), subacute (48 h-14 days), intermediate (14 days-6 months), and chronic (>6 months) [64]. To avoid using aggregated data in the subgroup analysis, age groups were generated by adding SDs to the mean; if the value is less than 60, the study is classified in the age group “Adults”, otherwise as “With Seniors”. Additionally, heterogeneity from serum vs. plasma sample differences for cytokines was tested. LOO and publication bias via Egger’s test and funnel plots were also performed. Subgroup analysis and meta-regression were conducted for immune markers with 10 or more studies, while LOO and publication bias were assessed for those with eight or more. All *p*-values < 0.05 were considered statistically significant. Statistical analyses were performed using dmetar package in R (version 4.2.2, R Foundation for Statitical Computing, Vienna, Austria).

## 5. Conclusions

Immune status among individuals with SCI is clinically influenced by the phase of injury, completeness of injury, sympathetic innervation impairment, age, and sex. Furthermore, the immune cell function is lower in individuals with SCI in the long term compared to those without SCI. Clinically, this information is vital for monitoring practices and tailoring management strategies to improve the immune status of individuals with SCI more effectively. By considering these factors in clinical decision-making, healthcare professionals can personalize management and infection monitoring that can improve the health outcomes for individuals with SCI.

## Figures and Tables

**Table 1:** Characteristics of studies included in the meta-analysis of the immune markers in blood, semen, and urothelium of individuals with and without SCI.

Sample	Blood (%)			Seminal Fluid (%)	Urothelium (%)
	Immune Cells ^a^	Cytokines ^a^	Immunoglobulins ^a^		
Number of studies	24	28	4	5	3
Total participants	1201	2089	309	253	110
With SCI	644	1180	178	149	80
Without SCI	557	909	131	104	30
Number of immune markers pooled	11	10	2	2	1
Mean age range for SCI (y)	26.9–57.0	26.9–54.9	40.6–55.6	30.4–35.0	37.9–48.2
Mean duration of injury range (y)	<1–24.0	<1–26.0	<1–18.7	4.7–10.5	^b^
Number of studies with disaggregated data on paraplegia	7 (30)	4 (14)	0	0	0
Number of studies with disaggregated data on tetraplegia	13 (57)	9 (32)	0	0	0
Number of studies in chronic phase	17 (74)	21 (75)	3 (75)	5 (100)	3 (100)
Number of studies with all males	11 (48)	14 (50)	2 (50)	5 (100)	0
Number of studies with disaggregated data on complete SCI	7 (30)	6 (21)	1 (25)	^c^	^c^
Number of studies with disaggregated data on above T6 injury	10 (43)	10 (36)	0	^c^	^c^
Number of studies > 30 sample size	11 (48)	13 (46)	3 (75)	2 (20)	2 (67)
Number of observational studies	18 (78)	28 (100)	4 (100)	5 (100)	0
Number of studies that used serum	-	14 (50)	2 (50)	-	-

^a^ Some studies have multiple immune markers that belong to multiple columns; ^b^ Only one study reported mean duration of injury, but all studies have individuals with chronic SCI; ^c^ Not reported; T6, 6th thoracic level; SCI, spinal cord injury; y, years.

**Table 2:** Summary of the random-effects meta-analysis of multiple immune markers in blood, seminal fluid, and the urothelium of individuals with and without SCI.

Immune Marker	Units	Pooled Studies (N)	SCI (N)	woSCI (N)	WMD	95% CI	I^2^ (%)	χ^2^
**Circulating Immune Cells**								
Leukocyte	1 × 10^9^/L	23	616	504	**0.78**	0.17, 1.38	83	<0.01
Neutrophil	1 × 10^9^/L	11	341	295	**0.76**	0.09, 1.42	89	<0.01
Lymphocyte	1 × 10^9^/L	14	387	334	−0.13	−0.27, 0.01	69	<0.01
Monocyte	1 × 10^9^/L	16	422	392	0.00	−0.03, 0.03	39	0.02
Eosinophil	1 × 10^9^/L	6	284	244	0.00	−0.04, 0.04	67	0.01
Basophil	1 × 10^9^/L	4	255	225	0.00	−0.01, 0.01	0	0.85
Granulocyte	1 × 10^9^/L	4	59	59	−0.21	−0.56, 0.14	39	0.18
B Cell	1 × 10^9^/L	2	23	23	0.01	−0.01, 0.03	0	0.73
T Cell	1 × 10^9^/L	2	23	23	0.02	−0.04, 0.62	55	0.11
NK Cell	1 × 10^9^/L	2	85	87	0.15	−0.32, 0.62	0	0.44
CD4/CD8	%	2	23	23	−0.03	−0.06, 0.00	0	0.95
**Circulating Cytokines and Chemokines**								
CRP	mg/L	12	549	415	**2.25**	1.14, 3.56	95	<0.01
hsCRP	mg/L	3	224	175	3.10	−1.00, 7.40	93	<0.01
CXCL1 ^a^	pg/mL	2	92	88	**40.09**	11.74, 68.45	0	0.93
CXCL1 ^b^	pg/mL	2	92	88	18.58	−24.42, 61.58	70	0.07
CXCL9 ^a^	pg/mL	2	100	89	**96.88**	36.68, 157.09	0	0.43
CXCL9 ^b^	pg/mL	2	90	89	−26.55	−26.55, 13.50	0	0.35
IL6 ^c^	pg/mL	13	484	363	**2.33**	1.20, 3.47	97	<0.01
IL6 ^d^	pg/mL	13	484	363	**0.89**	0.40, 1.37	83	<0.01
IL10	pg/mL	3	136	158	−0.14	−0.70, 0.42	0	0.63
MCP1	pg/mL	2	106	91	145.15	−166.12, 456.43	99	0
MIF	pg/mL	2	36	37	**798.51**	332.60, 1264.42	7	0.3
TGFβ	pg/mL	3	74	61	47.36	3539.26, 13,013.06	95	<0.01
TNFα	pg/mL	9	321	249	3.57	−2.22, 9.36	99	0
**Immunoglobulins**								
IgG	mg/mL	4	177	131	0.41	−2.01, 2.84	88	<0.01
IgM	mg/mL	2	81	41	**0.65**	0.26, 1.04	0	0.75
**Semen**								
Leukocyte	1 × 10^6^/L	3	123	80	**9.73**	5.21, 14.24	81	<0.01
Neutrophil	1 × 10^6^/L	2	26	24	**4.35**	2.37, 6.33	89	<0.01
**Urinary Tract**								
Mast Cells	positive/100 cells	3	80	30	**10.97**	4.08, 17.86	94	<0.01

^a,b^ The Hassanhashi et al. study has repeated measures, ^a^ after 48 h and ^b^ after 2 weeks for both the woSCI and SCI groups. ^c,d^ The de Mello Rieder et al. study measured IL6 at two time points, ^c^ was measured acutely and ^d^ subacutely. Bold values have WMD that is significantly different (*p*-value < 0.5). A positive value indicates a higher value among SCI vs. woSCI, while a negative value indicates lower value among SCI vs. woSCI. WMD, Weighted Mean Difference; woSCI, without spinal cord injury, CI, Confidence Interval; NK, natural killer; CD, cluster differentiation; CRP, C-reactive protein; hsCRP, high sensitivity CRP; CXCL, chemokine ligand; IL, interleukin; MCP, monocyte chemoattractant protein; MIF, migration inhibitory factor; TGF, transforming growth factor; TNF, tumor necrosis factor; IgG, immunoglobulin G; IgM, immunoglobulin M.

**Table 3:** Summary of the random-effects meta-analysis of the multiple immune markers found in the blood individuals with paraplegia or tetraplegia vs. those without SCI.

Immune Marker	Units	Pooled Studies (N)	SCI (N)	woSCI (N)	WMD	95% CI	I^2^ (%)	χ^2^
**Paraplegia Immune Markers**								
Leukocyte	1 × 10^9^/L	8	168	262	0.49	−0.88, 1.85	88	<0.01
Neutrophil	1 × 10^9^/L	6	151	246	0.85	−0.14, 1.84	93	<0.01
Lymphocyte	1 × 10^9^/L	7	159	254	−0.07	−0.27, 0.13	63	<0.01
Monocyte	1 × 10^9^/L	8	168	262	0.00	−0.06, 0.06	77	<0.01
Eosinophil	1 × 10^9^/L	4	132	215	0.00	−0.02, 0.02	0	0.75
Basophil	1 × 10^9^/L	3	126	209	0.00	−0.01, 0.01	0	1.00
Granulocytes	1 × 10^9^/L	2	20	33	−0.07	−1.53, 1.39	70	0.07
CRP	mg/dL	4	111	143	1.42	−0.58, 4.42	95	<0.01
IL6	pg/mL	3	99	120	0.75	−0.96, 2.46	94	<0.01
TNFa	pg/mL	2	57	91	−2.87	−9.51, 3.78	93	<0.01
**Tetraplegia Immune Markers**								
Leukocyte	1 × 10^9^/L	14	209	323	0.49	−0.17, 1.16	68	<0.01
Neutrophil	1 × 10^9^/L	7	138	262	0.83	−0.07, 1.73	92	<0.01
Lymphocyte	1 × 10^9^/L	12	193	307	−0.10	−0.23, 0.04	71	<0.01
Monocyte	1 × 10^9^/L	13	190	312	−0.01	−0.03, 0.02	14	0.30
Eosinophil	1 × 10^9^/L	5	117	231	0.00	−0.08, 0.09	93	<0.01
Basophil	1 × 10^9^/L	4	111	225	0.00	−0.01, 0.01	34	0.21
Granulocyte	1 × 10^9^/L	4	39	51	**−0.17**	−0.23, −0.11	0	0.51
B Cell	1 × 10^9^/L	2	15	15	0.01	−0.01, 0.03	0	0.51
T Cell	1 × 10^9^/L	2	15	15	−0.10	−0.12, 0.11	77	0.04
NK Cell	1 × 10^9^/L	2	15	15	−0.03	−0.06, 0.00	0	0.93
CD4/CD8	%	2	30	87	0.22	−0.38, 0.82	0	0.50
CRP	mg/dL	5	76	151	**14.18**	6.65, 21.70	92	<0.01
IL6	pg/mL	7	107	163	**0.87**	0.05, 1.69	81	<0.01
TNFα	pg/mL	2	35	91	−4.17	−13.28, 4.94	96	<0.01

Bold values have WMD that is significantly different (*p*-value < 0.5). A positive value indicates a higher value among SCI vs. nonSCI, while a negative value indicates lower value among SCI vs. nonSCI. WMD, Weighted Mean Difference; CI, Confidence Interval; NK, natural killer; CD, cluster differentiation; CRP, c-reactive protein; IL, interleukin; TNF, tumor necrosis factor; woSCI, without spinal cord injury.

**Table 4:** Summary of the changes in the functional capacity of immune cells in individuals with and without SCI at the acute-intermediate to chronic phases of injury.

Immune Cell	Changes at the Acute-Intermediate Phase vs. without SCI	Changes at the Chronic Phase vs. without SCI
Neutrophils	↑ oxidative capacity with increased expression of adhesion molecules acutely [20,21]	↓ phagocytic capacity among tetraplegics but not with paraplegics [22,23]
Monocytes	↑ oxidative capacity with increased expression of adhesion molecules acutely [20,21]	↓ expression of TLR4 and TLR9+ ↓ percentage that could phagocytose *Escherichia coli* [24]
Lymphocytes	↓ after 2 weeks but was restored after 3 months [25] ↔ oxidative capacity [20,21]	↔ no significant difference in activity [26] ↓ cytotoxicity [27]
Natural killer cells	↓ cell function [25]	↓ cell function *n* [25,28] ↔ no significant difference in cytoxicity [29,30,31]
Dendritic cells	-	↓ phenotypic maturation and significantly more pronounced in tetraplegia [32,33]

↓ significantly lower, ↑ significantly higher, ↔ no significant difference, - no data.

## Data Availability

Data are contained within the article and the Supplementary Materials.

## Supplementary Materials

The following supporting information can be downloaded at: https://www.mdpi.com/article/10.3390/ijms242216385/s1.
