# Supplementary material for: Immune Status of Individuals with Traumatic Spinal Cord Injury: A Systematic Review and Meta-Analysis
Source: Int J Mol Sci. 2023 Nov 16;24(22):16385. doi: 10.3390/ijms242216385 (PMC10670917; doi:10.3390/ijms242216385)
Supplement: Supplementary file 1 [file ijms-24-16385-s001.zip › ijms-2684783-supplementary.pdf]

List of materials provided in online supplement

Supplemental Appendix

Supplemental Appendix SI. Full Search Strategy

Supplemental Tables

Supplemental Table S1**.** Risk of bias of controlled studies in the immune status of individuals with spinal cord injury using the National Heart Lung and Blood Institute Quality Assessment Tool.

Supplemental Table S2. Risk of bias of observational studies in the immune status of individuals with spinal cord injury using the National Heart Lung and Blood Institute Quality Assessment Tool.

Supplemental Table S3. Summary of the studies included in the systematic review of the immune state difference of individuals with SCI vs individuals with woSCI and the changes in the immune status.

Supplemental Table S4. Summary of the studies that measured or studied circulating immune cell populations during the acute, subacute or intermediate phase among individuals with SCI compared to those without SCI.

Supplemental Table S5**.** Summary of the studies that measured or studied circulating immune cell populations during chronic phase among individuals with SCI compared to those without SCI.

Supplemental Table S6. Summary of the studies that measured circulating cytokines and chemokines during the acute, subacute and intermediate phase in individuals with SCI and compared to those without SCI.

Supplemental Table S7. Summary of studies that measured circulating cytokines and chemokines during the chronic phase among individuals with SCI and compared to those without SCI.

Supplemental Table S8**.** Summary of studies that compared immunoglobulins, serum antigens to diseases or circulating antibodies among individuals with SCI compared to those without SCI.

Supplemental Table S9. Summary of studies that compared immune markers in the seminal fluid among individuals with SCI compared to those without SCI.

Supplemental Table S10. Summary of studies that compared the immune markers in the urine and the urothelium among individuals with SCI compared to those without SCI.

Supplemental Table S11 Summary of studies that examined the immune markers in the bone marrow and saliva among individuals with SCI and compared them to those without SCI.

Supplemental Table S12. Summary of studies that investigate the circulating immune cells' functional activity and ability to mature, phagocytose or produce oxidative burst among individuals with SCI and compared to those without SCI.

Supplemental Table S13 Summary of the results of the subgroup analysis performed for the circulating immune markers that were included in the meta-analysis of studies comparing individuals with SCI vs those without SCI.

Supplemental Table S14. Summary of the results of the meta-regression analysis performed for the circulating immune markers that were included in the meta-analysis of studies comparing individuals with SCI vs those without SCI.

Supplemental Table S15. Summary of the Egger test results of circulating immune markers among individuals with SCI vs those without SCI

Supplemental Table S16. Summary of the Egger test results of circulating immune markers among individuals with tetraplegia vs those without SCI

Supplemental Figures

Supplemental Figure S1. The results of the leave one out analysis for the circulating leukocytes (A), monocytes (B), CRP (C), TNFα (D), IL6 (E), neutrophils (F), and lymphocytes (G) among the studies comparing individuals with SCI vs those without SCI.

Supplemental Figure S2. The results of the leave one out analysis for the circulating leukocytes (A), lymphocytes (B), and monocytes (C) among the studies comparing individuals with tetraplegia vs those without SCI.

Supplemental Figure S3. The funnel plot analysis for the circulating leukocytes (A), lymphocytes (B), monocytes (C), and neutrophils (D) among the studies comparing individuals with SCI vs those without SCI.

Supplemental Figure S4. The funnel plot analysis for the circulating CRP (A), IL6 (B), and TNFα (C) among the studies comparing individuals with SCI vs those without SCI.

Supplemental Figure S5. The funnel plot analysis for the circulating leukocytes (A), lymphocytes (B), and monocytes (C) among the studies comparing individuals with tetraplegia vs those without SCI.

Supplemental Appendix SII. Full Search Strategy
Overview databases & results
Date last searched **11.01.2023**

	Before deduplication	After deduplication
Embase.com	3982	3829
Medline Ovid	1274	811
PubMed	6833	5211
Cochrane CENTRAL	232	186
Total	12321	10037

2284 duplicates

PUBMED
(Spinal Cord Injuries[mh] OR Spinal Cord Ischemia[mh] OR Paraplegia[mh] OR Quadriplegia[mh] OR (((spine[tiab] OR spinal[tiab]) AND (injur*[tiab] OR trauma*[tiab] OR damag*[tiab])) OR (spinal cord[tiab] AND (disease*[tiab] OR contusion*[tiab] OR laceration*[tiab] OR transection*[tiab] OR lesion*[tiab] OR trauma*[tiab] OR ischemi*[tiab] OR ischaemi*[tiab])) OR (myelopath*[tiab] AND (trauma*[tiab] OR post-trauma*[tiab] OR posttrauma*[tiab])) OR ((spine[tiab] OR spinal[tiab] OR vertebra*[tiab]) AND (fracture*[tiab] OR trauma*[tiab] OR injur*[tiab] OR damage*[tiab] OR wound*[tiab])) OR SCI-group*[tiab] OR paraplegi*[tiab] OR quadriplegi*[tiab] OR tetraplegi*[tiab]))
AND
(("Immune System"[Mesh]) OR "Immune System Phenomena"[Mesh] OR ("Allergy and Immunology"[Mesh]) OR "immunology" [Subheading] OR ("Neuroglia"[Mesh])) OR ((immun*[tiab] or cytokine*[tiab] or chemokine*[tiab] or antibod*[tiab] or interleukin*[tiab] or antigen*[tiab] or lymph*[tiab] or HLA[tiab] or MHC[tiab] or complement activation[tiab] or antibody-dependent cell cytotoxicity[tiab] or macrophage activation[tiab] or neutrophil*[tiab] or phagocytosis[tiab] or macrophage*[tiab] or seroconversion[tiab] or th1-th2 balance or dendritic[tiab] or eosinophil*[tiab] or basophil* [tiab] or megakaryocyte*[tiab] or monocyte*[tiab] or leukocyte*[tiab] or granulocyte*[tiab] or megakaryocyte*[tiab] or erythrocyte*[tiab] or mast cells[tiab] or platelet*[tiab] or B cell[tiab] or T cell[tiab] or natural killer cells[tiab] or antigen presenting cells[tiab]))
AND
("Humans"[MeSH Terms] OR "human*"[Title/Abstract]) AND (("Adult"[Mesh]) OR "Young Adult"[Mesh] OR adult*[tiab])
NOT
((animals[mh] NOT humans[mh])) OR ((letter[pt] OR news[pt] OR comment[pt] OR editorial[pt] OR congress[pt]))

MEDLINE
exp Immune System/ OR exp Immune System Phenomena/ OR exp "Allergy and Immunology"/ OR exp Immunoproteins/ OR (immun*).ab,ti. AND (cytokine* or chemokine* or antibod* or interleukin* or antigen* or lymph* or major histocompatibility complex or complement* or neutrophil* or phagocytosis or macrophage* or dendritic cells or eosinophil* or basophil* or megakaryocyte* or monocyte* or leukocyte* or granulocyte* or megakaryocyte* or erythrocyte* or mast cells or platelet* or B cell or T cell or natural killer cells or antigen presenting cells or hs-crp or crp).ab,ti.
AND
exp Spinal Cord Injuries/ OR exp Spinal Cord Ischemia/ OR exp Paraplegia/ OR
exp Quadriplegia/ OR (((spine or spinal) adj3 (injur* or trauma* or damag*)) or (spinal cord adj3 (disease* or contusion* or laceration* or transection* or lesion* or trauma* or ischemi* or ischaemi*)) or (myelopath* adj3 (trauma* or post-trauma* or posttrauma*)) or ((spine or spinal or vertebra*) adj3 (fracture* or trauma* or injur* or damage* or wound*)) or paraplegi* or quadriplegi* or tetraplegi*).ab,ti.
AND
(exp Adult/ OR exp Young Adult/) OR adult*.ab,ti. AND (exp Humans/) or human*.ab,ti.
NOT
(letter or news or comment or editorial or congres* or abstract*).pt.

EMBASE
(exp Immune System/ OR exp Immune System Phenomena/ OR exp "Allergy and Immunology"/) OR (immun*or cytokine* or chemokine* or antibod* or interleukin* or antigen* or lymph* or HLA or MHC or complement activation or antibody-dependent cell cytotoxicity or macrophage activation or neutrophil* or phagocytosis or macrophage* or seroconversion or th1-th2 balance or dendritic or eosinophil* or basophil* or megakaryocyte* or monocyte* or leukocyte* or granulocyte* or megakaryocyte* or erythrocyte* or mast cells or platelet* or B cell or T cell or natural killer cells).ab,ti.
AND
exp Spinal Cord Injuries/ OR exp Spinal Cord Ischemia/ OR exp Paraplegia/ OR exp Quadriplegia/ OR (((spine or spinal) adj3 (injur* or trauma* or damag*)) or (spinal cord adj3 (disease* or contusion* or laceration* or transection* or lesion* or trauma* or ischemi* or ischaemi*)) or (myelopath* adj3 (trauma* or post-trauma* or posttrauma*)) or ((spine or spinal or vertebra*) adj3 (fracture* or trauma* or injur* or damage* or wound*)) or paraplegi* or quadriplegi* or tetraplegi*).ab,ti.
AND

(exp Adult/ OR exp Young Adult/) OR adult*.ab,ti.
NOT
((letter or news or comment or editorial or congres* or abstract*).pt. OR (exp animals/ not humans/))

COCHRANE
Immune System OR Immune System Phenomena OR "Allergy and Immunology" OR (immun* OR cytokine* OR chemokine* OR antibod* OR interleukin* OR antigen* OR lymph* OR HLA OR MHC OR complement activation OR antibody-dependent cell cytotoxicity OR macrophage activation OR neutrophil* OR phagocytosis OR macrophage* OR seroconversion OR th1 th2 balance OR dendritic OR eosinophil* OR basophil* OR megakaryocyte* OR monocyte* OR leukocyte* OR granulocyte* OR megakaryocyte* OR erythrocyte* OR mast cells OR platelet* OR B cell OR T cell OR natural killer cells OR antigen presenting cells OR astrocyte* OR oligodenro* OR microglia):ab,ti,kw
AND
pinal Cord Injuries OR Spinal Cord Ischemia OR Paraplegia OR Quadriplegia OR ((((spine or spinal) NEAR/3 (injur* or trauma* or damag*)) OR ('spinal cord' NEAR/3 (disease* or contusion* or laceration* or transection* or lesion* or trauma* or ischemi* or ischaemi*)) OR (myelopath* NEAR/3 (trauma* or post-trauma* or posttrauma*)) OR ((spine or spinal or vertebra*) NEAR/3 (fracture* or trauma* or injur* or damage* or wound*)) OR SCI-group* OR 'central cord injury syndrome' OR 'central cord syndrome' OR 'central spinal cord syndrome' OR 'cauda equine syndrome' OR 'anterior cord syndrome' OR 'conus medullaris syndrome' OR 'Brown Sequard' OR paraplegi* OR quadriplegi* OR tetraplegi*):ab,ti,kw)
AND
(Adult OR Young Adult) OR (adult*):ab,ti,kw

Supplemental Table S1**.** Risk of bias of controlled studies in the immune status of individuals with spinal cord injury using the National Heart Lung and Blood Institute Quality Assessment Tool.

Lead Author, Publication (Supplemental Reference); Year	Study Design	1	2	3	4	5	6	7	8	9	10	11	12	13	14	Risk of bias (Total score)[1]	Quality Rating Scheme for Studies and Other Evidence
Ueta 2008 [1]	nRCT	N	NA	N	N	N	Y	Y	Y	Y	Y	Y	N	NR	NA	6/12 (50%)	2
Szymczak, 2021 [2]	nRCT	N	NA	N	N	N	N	Y	Y	Y	Y	Y	N	NR	NA	5/12 (42%)	2
Hashizaki, 2018 [3]	nRCT	N	NA	N	N	N	Y	Y	Y	Y	Y	Y	N	NR	NA	6/12 (50%)	2
Umemoto, 2011 [4]	nRCT	N	NA	N	N	N	Y	Y	Y	Y	Y	Y	N	NR	NA	6/12 (50%)	2
Kouda, 2012 [5]	nRCT	N	NA	N	N	N	Y	Y	Y	Y	Y	Y	N	NR	NA	6/12 (50%)	2
Yamanaka, 2004 [6]	nRCT	N	NA	N	N	N	Y	Y	Y	Y	Y	Y	N	NR	NA	6/12 (50%)	2
Chen, 2016 [7]	nRCT	N	NA	N	N	N	Y	Y	Y	Y	Y	Y	N	NR	NA	6/12 (50%)	2
Chen, 2016 [8]	nRCT	N	NA	N	N	N	Y	Y	Y	Y	Y	Y	N	NR	NA	6/12 (50%)	2
Jiang, 2015 [9]	nRCT	N	NA	N	N	N	Y	Y	Y	Y	Y	Y	N	NR	NA	6/12 (50%)	2
Leicht, 2015 [10]	nRCT	N	NA	N	N	N	NR	Y	Y	Y	Y	Y	N	NR	NA	5/12 (42%)	2
Leicht, 2011 [11]	nRCT	N	NA	N	N	N	N	Y	Y	Y	Y	Y	N	NR	NA	5/12 (42%)	2

Abbreviations: CD, cannot determine; NA, not applicable; NR, not reported
[1]Risk of bias rating (Low (75-100%), Moderate (50-74%), or High (0-49%))

Criteria used to assess risk of bias of controlled clinical trials:

1. Was the study described as randomized, a randomized trial, a randomized clinical trial, or an RCT?
2. Was the method of randomization adequate (i.e., use of randomly generated assignment)?
3. Was the treatment allocation concealed (so that assignments could not be predicted)?
4. Were study participants and providers blinded to treatment group assignment?
5. Were the people assessing the outcomes blinded to the participants' group assignments?
6. Were the groups similar at baseline on important characteristics that could affect outcomes (e.g., demographics, risk factors, co-morbid conditions)?

7. Was the overall drop-out rate from the study at endpoint 20% or lower of the number allocated to treatment?
8. Was the differential drop-out rate (between treatment groups) at endpoint 15 percentage points or lower?
9. Was there high adherence to the intervention protocols for each treatment group?
10. Were other interventions avoided or similar in the groups (e.g., similar background treatments)?
11. Were outcomes assessed using valid and reliable measures, implemented consistently across all study participants?
12. Did the authors report that the sample size was sufficiently large to be able to detect a difference in the main outcome between groups with at least 80% power?
13. Were outcomes reported or subgroups analyzed prespecified (i.e., identified before analyses were conducted)?
14. Were all randomized participants analyzed in the group to which they were originally assigned, i.e., did they use an intention-to-treat analysis?

Criteria used to assess risk of bias of pre-post study without control group:
1. Was the study question or objective clearly stated?
2. Were eligibility/selection criteria for the study population prespecified and clearly described?
3. Were the participants in the study representative of those who would be eligible for the test/service/intervention in the general or clinical population of interest?
4. Were all eligible participants that met the prespecified entry criteria enrolled?
5. Was the sample size sufficiently large to provide confidence in the findings?
6. Was the test/service/intervention clearly described and delivered consistently across the study population?
7. Were the outcome measures prespecified, clearly defined, valid, reliable, and assessed consistently across all study participants?

8. Were the people assessing the outcomes blinded to the participants' exposures/interventions?
9. Was the loss to follow-up after baseline 20% or less? Were those lost to follow-up accounted for in the analysis?
10. Did the statistical methods examine changes in outcome measures from before to after the intervention? Were statistical tests done that provided p values for the pre-to-post changes?
11. Were outcome measures of interest taken multiple times before the intervention and multiple times after the intervention (i.e., did they use an interrupted time-series design)?
12. If the intervention was conducted at a group level (e.g., a whole hospital, a community, etc.) did the statistical analysis take into account the use of individual-level data to determine effects at the group level?

Supplemental Table S2. Risk of bias of observational studies in the immune status of individuals with spinal cord injury using the National Heart Lung and Blood Institute Quality Assessment Tool.

Lead Author, Publication (Supplemental Reference); Year	Study Design	1	2	3	4	5	6	7	8	9	10	11	12	13	14	Risk of bias (Total score)[1]	Quality Rating Scheme for Studies and Other Evidence
Kliesch, 1996 [12]	Cohort	Y	Y	NR	Y	N	Y	N	Y	Y	NA	Y	NR	Y	N	8/13 (61%)	2
Wichmann, 2022 [13]	Cohort	Y	Y	NR	Y	N	Y	N	Y	Y	NA	Y	NR	Y	Y	9/13 (69%)	2
Furlan, 2006 [14]	Case Control	Y	Y	N	NR	Y	Y	Y	Y	Y	Y	NR	N	NA	NA	8/12 (66%)	3
Heller, 2017 [15]	Cohort	Y	Y	NR	Y	N	Y	N	Y	Y	NA	Y	NR	Y	N	8/13 (61%)	2
Aird, 1999 [16]	Cross-sectional	Y	Y	NR	Y	N	Y	Y	Y	Y	NA	Y	NR	NA	N	8/12 (67%)	4
Basu, 2002 [17]	Cross-sectional	Y	Y	NR	Y	N	Y	Y	Y	Y	NA	Y	Y	NA	N	9/12 (75%)	4
Brackett, 2008 [18]	Cross-sectional	Y	Y	NR	Y	N	Y	Y	Y	Y	NA	Y	Y	NA	N	9/12 (75%)	4
Brackett, 2007 [19]	Cross-sectional	Y	Y	NR	Y	N	Y	Y	Y	Y	NA	Y	Y	NA	N	9/12 (75%)	4
da Silva, 2010 [20]	Cross-sectional	Y	Y	NR	Y	N	Y	Y	Y	Y	NA	Y	Y	NA	N	9/12 (75%)	4
da Silva, 2013 [21]	Cross-sectional	Y	Y	NR	NR	N	Y	Y	Y	Y	NA	Y	Y	NA	N	8/12 (67%)	4
Padron, 1997 [22]	Cross-sectional	Y	Y	NR	NR	N	Y	Y	Y	N	NA	Y	NR	NA	N	6/12 (50%)	4
Salsabili, 2006 [23]	Cross-sectional	Y	Y	NR	Y	N	Y	Y	Y	Y	NA	Y	Y	NA	N	9/12 (75%)	4
Trabulsi, 2002 [24]	Cross-sectional	Y	Y	NR	NR	N	Y	Y	Y	Y	NA	Y	NR	NA	N	8/12 (67%)	4
Basu, 2004 [25]	Cross-sectional	Y	Y	NR	Y	N	Y	Y	Y	Y	NA	Y	Y	NA	N	9/12 (75%)	4
Zhang, 2013 [26]	Cross-sectional	Y	Y	NR	Y	N	Y	Y	Y	Y	NA	Y	NR	NA	N	9/12 (75%)	4
Bao, 2011 [27]	Cross-sectional	Y	Y	NR	Y	N	Y	N	Y	Y	NA	Y	NR	NA	N	7/12 (58%)	4
Bao, 2009 [28]	Cohort	Y	Y	NR	Y	N	Y	N	Y	Y	NA	Y	NR	Y	N	8/13 (53%)	2
Lin, 2021 [29]	Cross-sectional	Y	Y	NR	Y	N	Y	N	Y	Y	NA	Y	NR	NA	N	7/12 (58%)	4
Riegger, 2009 [30]	Cohort	Y	Y	NR	Y	N	Y	Y	Y	Y	NA	Y	NR	Y	N	9/13 (69%)	2
Heller, 2021 [31]	Cohort	Y	Y	NR	Y	N	Y	N	Y	Y	NA	Y	NR	Y	N	8/13 (61%)	2
Xu, 2019 [32]	Cross-sectional	Y	Y	NR	Y	N	Y	N	Y	Y	NA	Y	NR	NA	N	7/12 (58%)	4
Paczkowska, 2015 [33]	Cross-sectional	Y	Y	NR	N	N	Y	N	Y	Y	NA	Y	NR	NA	N	6/12 (50%)	4
Shi, 2022 [34]	Cross-sectional	Y	Y	NR	Y	N	Y	N	Y	Y	NA	Y	NR	NA	N	7/12 (58%)	4
Arevalo-Martin, 2018 [35]	Cross-sectional	Y	Y	NR	Y	N	Y	N	Y	Y	NA	Y	NR	NA	N	7/12 (58%)	4
Grassner, 2022 [36]	Cohort	Y	Y	NR	Y	N	Y	Y	Y	Y	NA	Y	NR	Y	Y	10/13 (76%)	3
Campagnolo, 1994 [37]	Cross-sectional	Y	Y	NR	NR	N	Y	Y	Y	Y	NA	Y	NR	NA	N	7/12 (58%)	4
Campagnolo, 1997 [38]	Cross-sectional	Y	Y	NR	N	N	Y	Y	Y	Y	NA	Y	NR	NA	N	7/12 (58%)	4
Campagnolo, 2008 [39]	Cross-sectional	Y	Y	NR	Y	N	Y	Y	Y	Y	NA	Y	Y	NA	Y	10/12 (83%)	4
Davies, 2007 [40]	Cross-sectional	Y	N	NR	Y	N	Y	N	N	Y	NA	Y	NR	NA	Y	6/12 (50%)	4
Diaz, 2021 [41]	Cross-sectional	Y	Y	NR	Y	N	Y	Y	Y	Y	NA	Y	NR	NA	Y	9/12 (75%)	4
Formisano, 1998 [42]	Cross-sectional	Y	N	NR	Y	N	Y	N	Y	Y	NA	Y	NR	NA	N	6/12 (50%)	4
Gucluler, 2017 [43]	Cross-sectional	Y	Y	NR	N	N	Y	Y	Y	Y	NA	Y	NR	NA	N	7/12 (58%)	4
Kanyilmaz, 2013 [44]	Cross-sectional	Y	Y	NR	NR	N	Y	Y	Y	Y	NA	Y	NR	NA	N	7/12 (58%)	4

Hayes, 2002 [45]	Cross-sectional	Y	Y	NR	NR	N	Y	Y	Y	Y	NA	Y	NR	NA	N	7/12 (58%)	4
Pan, 2005 [46]	Case Control	Y	Y	N	NR	N	NR	Y	Y	Y	Y	NR	N	NA	NA	6/12 (50%)	3
Monahan, 2015 [47]	Cross-sectional	Y	Y	NR	Y	N	Y	Y	Y	Y	NA	Y	NR	NA	N	8/12 (67%)	4
Pavlicek, 2017 [48]	Cross-sectional	Y	Y	NR	Y	N	Y	Y	Y	Y	NA	Y	NR	NA	Y	9/12 (75%)	4
Iversen, 2004 [49]	Cross-sectional	Y	Y	NR	NR	N	Y	Y	Y	Y	NA	Y	NR	NA	N	7/12 (58%)	4
Hsieh, 2009 [50]	Cross-sectional	Y	Y	NR	Y	N	Y	Y	Y	Y	NA	Y	NR	NA	Y	9/12 (75%)	4
Pang, 2022 [51]	Cross-sectional	Y	Y	NR	Y	N	Y	N	Y	Y	NA	Y	NR	NA	Y	8/12(67%)	4
Bank, 2015 [52]	Cohort	Y	Y	NR	N	N	Y	N	Y	Y	NA	Y	N	N	N	6/13 (46%)	2
Huang, 2014 [53]	Cross-sectional	Y	Y	NR	Y	N	Y	N	Y	Y	NA	Y	N	NA	N	7/12 (58%)	4
Yong, 2018 [54]	Cross-sectional	Y	Y	NR	Y	N	Y	Y	Y	Y	NA	Y	NR	NA	N	8/12 (67%)	4
de Mello Rieder, 2018 [55]	Cohort	Y	Y	N	Y	Y	Y	N	Y	Y	Y	NA	N	Y	N	9/13 (69%)	2
Liu, 2005 [56]	Cross-sectional	Y	N	NR	Y	N	Y	N	Y	Y	NA	Y	NR	NA	Y	7/12 (58%)	4
Wang, 2007 [57]	Cross-sectional	Y	Y	NR	Y	N	Y	Y	Y	Y	NA	Y	NR	NA	Y	9/12 (75%)	4
Stein, 2013 [58]	Cross-sectional	Y	Y	NR	Y	N	Y	Y	Y	Y	NA	Y	NR	NA	N	8/12 (67%)	4
Chen, 2020 [59]	Cross-sectional	Y	Y	NR	Y	N	Y	N	Y	Y	NA	Y	NR	NA	N	7/12 (58%)	4
Chen, 2020 [60]	Cross-sectional	Y	Y	NR	Y	N	Y	N	Y	Y	NA	Y	NR	NA	N	7/12 (58%)	4
Matos-Souza, 2010 [61]	Cross-sectional	Y	Y	NR	Y	N	Y	Y	Y	Y	NA	Y	NR	NA	N	8/12 (67%)	4
Mi, 2019 [62]	Cross-sectional	Y	Y	NR	Y	N	Y	Y	Y	Y	NA	Y	NR	NA	N	8/12 (67%)	4
Segal, 1993 [63]	Cross-sectional	Y	Y	NR	NR	N	Y	NA	Y	Y	NA	Y	NR	NA	N	6/11 (54%)	4
Baria, 2021 [64]	Cross-sectional	Y	Y	NR	Y	N	Y	Y	Y	Y	NA	Y	NR	NA	N	8/12 (72%)	4
Rogeri, 2005 [65]	Cross-sectional	Y	Y	NR	Y	N	Y	Y	Y	Y	NA	Y	NR	NA	N	8/12 (67%)	4
Frost, 2005 [66]	Cross-sectional	Y	Y	NR	Y	N	Y	Y	Y	Y	NA	Y	NR	NA	Y	9/12 (75%)	4
Radulovic, 2015 [67]	Cross-sectional	Y	Y	NR	Y	N	Y	Y	Y	Y	NA	Y	NR	NA	N	8/12 (72%)	4
Liang, 2008 [68]	Cross-sectional	Y	Y	NR	Y	N	Y	Y	Y	Y	NA	Y	NR	NA	Y	9/12 (75%)	4
Wang, 2009 [69]	Cross-sectional	Y	Y	NR	Y	N	Y	Y	Y	Y	NA	Y	NR	NA	Y	9/12 (75%)	4
Brewster, 2020 [70]	Cross-sectional	Y	Y	NR	Y	N	Y	Y	Y	Y	NA	Y	NR	NA	N	8/12 (67%)	4
Palmers, 2016 [71]	Cohort	Y	Y	NR	Y	N	Y	N	Y	Y	NA	Y	NR	Y	N	8/13 (61%)	2
Shnawa, 2020 [72]	Cross-sectional	Y	Y	NR	Y	N	Y	Y	Y	Y	NA	Y	NR	NA	Y	9/12 (75%)	4
Chinigo, 1996 [73]	Cross-sectional	Y	Y	NR	Y	N	Y	Y	Y	Y	NA	Y	NR	NA	N	8/12 (67%)	4
Everall, 1974 [74]	Cross-sectional	Y	N	NR	NR	N	Y	Y	Y	Y	NA	N	NR	NA	N	5/12 (41%)	4
Trautner, 2004 [75]	Case Control	Y	Y	N	Y	Y	NR	Y	Y	Y	Y	NR	N	NA	NA	8/12 (67%)	3
Rosman, 1998 [76]	Cross-sectional	Y	N	NR	Y	N	Y	Y	Y	Y	NA	Y	NR	NA	N	7/12 (58%)	4
Lynch, 2002 [77]	Case Control	Y	Y	N	NR	Y	NR	Y	NR	Y	Y	NR	N	NA	NA	6/12 (50%)	3
Vaidyanathan, 2000 [78]	Cross-sectional	Y	Y	NR	NR	N	Y	N	Y	N	NA	N	NR	NA	N	4/12 (25%)	4
Gallego Gomez, 1983 [79]	Cross-sectional	Y	N	NR	Y	N	Y	N	Y	N	NA	Y	N	NA	Y	6/12 (50%)	4
Fraussen, 2022 [80]	Cross-sectional	Y	Y	NR	Y	N	Y	N	Y	Y	NA	Y	N	NA	Y	8/12 (67%)	4

Chernykh, 2006 [81]	Cross-sectional	Y	Y	NR	NR	N	Y	Y	Y	Y	NA	Y	NR	NA	N	7/12 (58%)	4
Iversen, 2000 [82]	Cross-sectional	Y	Y	NR	NR	N	Y	Y	Y	Y	NA	Y	NR	NA	N	7/12 (58%)	4
Hassanshahi, 2013 [83]	Cohort	Y	Y	NR	Y	N	Y	N	Y	Y	NA	Y	NR	Y	N	8/13 (61%)	2
Lieberman, 2014 [84]	Cross-sectional	Y	Y	NR	Y	N	Y	Y	Y	Y	NA	Y	NR	NA	N	8/12 (67%)	4
La Favor, 2011 [85]	Cross-sectional	Y	Y	NR	Y	N	Y	Y	Y	Y	NA	Y	NR	NA	N	8/12 (67%)	4
Edwards, 2008 [86]	Cross-sectional	Y	Y	NR	N	N	Y	Y	Y	Y	NA	Y	NR	NA	N	7/12 (58%)	4
Iversen, 2002 [87]	Cohort	Y	Y	NR	Y	N	Y	Y	Y	Y	NA	Y	NR	Y	N	9/13 (69%)	2
Invernizzi, 2015 [88]	Cross-sectional	Y	Y	NR	Y	N	Y	Y	Y	Y	NA	Y	NR	NA	N	8/12 (67%)	4
Schreiber, 2017 [89]	Cross-sectional	Y	Y	NR	Y	N	Y	Y	Y	Y	NA	Y	NR	NA	N	8/12 (67%)	4
Bernardi, 2019 [90]	Cross-sectional	Y	Y	NR	Y	N	Y	Y	N	Y	NA	Y	NR	NA	N	7/12 (58%)	4

Abbreviations: CD, cannot determine; NA, not applicable; NR, not reported

¹ Risk of bias rating (Low (75-100%), Moderate (50-74%), or High (0-49%))

Criteria used to asses risk of bias of cohort/cross-sectional studies:

1. Was the research question or objective in this paper clearly stated?
2. Was the study population clearly specified and defined?
3. Was the participation rate of eligible persons at least 50%?
4. Were all the subjects selected or recruited from the same or similar populations (including the same time period)? Were inclusion and exclusion criteria for being in the study prespecified and applied uniformly to all participants?
5. Was a sample size justification, power description, or variance and effect estimates provided?
6. For the analyses in this paper, were the exposure(s) of interest measured prior to the outcome(s) being measured?
7. Was the timeframe sufficient so that one could reasonably expect to see an association between exposure and outcome if it existed?
8. For exposures that can vary in amount or level, did the study examine different levels of the exposure as related to the outcome (e.g., categories of exposure, or exposure measured as continuous variable)?
9. Were the exposure measures (independent variables) clearly defined, valid, reliable, and implemented consistently across all study participants?
10. Was the exposure(s) assessed more than once over time?
11. Were the outcome measures (dependent variables) clearly defined, valid, reliable, and implemented consistently across all study participants?
12. Were the outcome assessors blinded to the exposure status of participants?
13. Was loss to follow-up after baseline 20% or less?
14. Were key potential confounding variables measured and adjusted statistically for their impact on the relationship between exposure(s) and outcome(s)?

Criteria used to asses risk of bias of case-control studies:

1. Was the research question or objective in this paper clearly stated and appropriate?
2. Was the study population clearly specified and defined?
3. Did the authors include a sample size justification?
4. Were controls selected or recruited from the same or similar population that gave rise to the cases (including the same timeframe)?
5. Were the definitions, inclusion and exclusion criteria, algorithms or processes used to identify or select cases and controls valid, reliable, and implemented consistently across all study participants?
6. Were the cases clearly defined and differentiated from controls?
7. If less than 100 percent of eligible cases and/or controls were selected for the study, were the cases and/or controls randomly selected from those eligible?
8. Was there use of concurrent controls?
9. Were the investigators able to confirm that the exposure/risk occurred prior to the development of the condition or event that defined a participant as a case?
10. Were the measures of exposure/risk clearly defined, valid, reliable, and implemented consistently (including the same time period) across all study participants?
11. Were the assessors of exposure/risk blinded to the case or control status of participants?
12. Were key potential confounding variables measured and adjusted statistically in the analyses? If matching was used, did the investigators account for matching during study analysis?

Supplemental Table S3. Summary of the studies included in the systematic review of the immune state difference of individuals with SCI vs individuals with woSCI and the changes in the immune status.

Sample source	Immune markers	Phase of SCI	Total number of included studies (N) [Supplemental Table]	Included studies in the meta-analysis (N)
Blood	*Circulating Immune Cells*<br>Leukocytes, Neutrophil, Monocytes, Lymphocytes, Basophils, Eosinophils, CD14+monocytes, CD3+T lymphocytes, MHC class II (HLA-DR), CD15+ granulocytes, CD19+ B lymphocytes, CD56, CD4+ Tcells, CD8+ Tcells, B cells, NK cells, Natural killer cell function	Acute-Subacute-Intermediate<br><br>Chronic	*SCI vs woSCI (13)*<br>[Supplemental Table 4]<br><br>*SCI vs woSCI (27)*<br>[Supplemental Table 5]	*(5)*<br><br>*(18)*
	Circulating Cytokines and Chemokines<br>b-FGF, CTACK, eotaxin, G-CSF, GM-CSF, GRO-a/CXCL1, HGF, IFN-a2, IFN-y, IL-1a, IL-1b, IL-1Ra, IL-2, IL-2Ra, IL-3, IL-4, IL-5, IL-6, IL-7, IL-8, IL-9, IL-10, IL-12p40, IL-12p70, IL-13, IL-15, IL-16, IL-17, IL-18,IL37, IP-10, LIF, MCP-1/CCL2, MCP-3/CCL7, MCSF, MIF, MIG/CXCL9, MIP1-a/ CCL3, MIP1-b/CCL4, b-NGF, PDGF-bb, RANTES/CCR1, SCF, SCGF-b, SDF1-a, TNF-a, TNF-b, CCL21, CXCL13, CCL27, CXCL5, CCL11, CCL24, CCL26, CX3CL1, CXCL6, GMCSF, CXCL1, CXCL2, CCL1, MCP-2, MCP-3, MCP-4, CCL22, MIP-3a, MIP-3b, MPIF-1, CXCL16, CXCL12, CCL17, CCL25	Acute-Subacute-Intermediate<br><br>Chronic	*SCI vs woSCI (14)*<br>[Supplemental Table 6]<br><br>*SCI vs woSCI (28)*<br>[Supplemental Table 7]	*(6)*<br><br>*(22)*
	Antibodies and Antigens<br>IgG, IgM, IgA<br>CMV antigen, HbSAg		*SCI vs woSCI (10)*<br>[Supplemental Table 8]	*(3)*
Semen	Mononuclear cells, CD34+(%, counts, G0/G1 %, S/G2M%, apoptosis%), CD34+CD38-(%), Percentages of lymphocyte subpopulations, NK cells, T cells, B cell, colony formation of progenitor cells, CD3, CD19, CD16/56, Leukocytes, Neutrophil, Monocytes, Lymphocytes, Eosinophils	Chronic	*SCI vs woSCI (11)*<br>[Supplemental Table 9]	*(5)*
Urine and urothelium	Mast cells, IgA, IgG, Leukocytes, Lymphocytes, Neutrophils	Chronic	*SCI vs woSCI (7)*<br>[Supplemental Table 10]	*(3)*
Bone Marrow And Saliva	Leukocytes, Neutrophils, Monocytes, Lymphocytes, Colony formation of progenitor cells, Salivary IgA	Chronic	*SCI vs woSCI (4)*<br>[Supplemental Table 9]	(-)

Supplemental Table S4. Summary of the studies that measured or studied circulating immune cell populations during the acute, subacute or intermediate phase among individuals with SCI compared to those without SCI.

Lead Author	SCI (n)	woSCI (n)	Male (%)	Age (sd) [range]	Day duration of injury [range]	SCI Phase	SCI Population characteristics	Sample	Immunological markers used	Significantly Increased Markers	Significantly Decreased Markers	Other significant finding	Risk of bias (Quality of Evidence Rating)
Bao, 2011, Bao, 2009 Canada [27,28]	9 C:3 T:3 L:3	16	SCI: 7 (78) woSCI: 12 (75)	SCI: 44 (20.4) woSCI: 39.9 (13.1)	[0-14]	Acute-Subacute	Healthy adults	Blood (Plasma)	Leukocytes, neutrophil, monocytes, lymphocytes	Neutrophils, monocytes		Oxidative capacity of neutrophils and monocytes was significantly increased compared to trauma controls. Lymphocytes had no evidence of oxidative burst activity. Difference in beta-integrins expression between trauma control and SCI after 24-48hrs injury.	Moderate (4,2)
Furlan, 2006 [14]	21 C:21	11	SCI: 25 (71) woSCI:6 (55)	SCI: 57 [17-83] woSCI: 41 [18-75]	[0-7]	Acute-Subacute	Healthy adults	Blood	Leukocyte, Lymphocytes	Leukocyte, Lymphocytes		Leukocyte and lymphocyte counts did not significantly vary up to 7 days post injury	Moderate (3)
Lin, 2021 China [29]	114 Pp: 63 Tp: 51	90	SCI: 83 (72) woSCI:48 (53)	SCI: 55.2 (12.7) woSCI: 45.1 (10.2)	[7-21]	Subacute-Intermediate	Healthy adults	Blood	Leukocytes, Neutrophils, Lymphocytes, Monocytes, Eosinophils, Basophils	Leukocytes, Neutrophils, Monocytes			Moderate (4)
Riegger, 2009 Germany [30]	16 C:6 T:8 L:2	10	SCI: 14 (88) woSCI: 7 (70)	SCI: 37.9 (13.4) woSCI: 51.6 (15.8)	[1-136]	Acute-Subacute-Intermediate	Healthy adults	Blood (Plasma)	CD14+monocytes, CD3+T lymphocytes, MHC class II (HLA-DR), CD15+ granulocytes, CD19+ B lymphocytes	CD15+ granulocytes (after 24hrs)	CD14+ monocytes, CD3+ T lymphocyte, CD19+ B lymphocytes, MHC classII+ cells (after 24hrs)	No significant difference after 6-8 days with controls in all cell types tested after 136 days.	Moderate (2)

Heller, 2021 Germany [31]	18	4	SCI: 15 (83) woSCI: 4 (100)	SCI: 49.7 (39.0) woSCI: 43 (median)	[0-90]	Acute-Subacute-Intermediate	Healthy adults	Blood (Plasma)	Monocyte (CD14, CD16, IL10, CXCL4), Lymphocyte transformation			Classical monocytes were lower among those with neurological remission vs to those who did not. Non-classical monocytes were lower among SCI vs control. Total lymphocytes had similar frequency compared to woSCI. Those with neurological remission had lymphocytes response recovered to elevated levels compared to non-remission group. Early elevated concentrations of CD14-/CD16+/IL10+/CXCR4int monocytes were elevated and related to higher odds of CNS regeneration.	Moderate (2)
Xu, 2019 China [32]	21 C:21	20	SCI: 15 (71) woSCI: 11(55)	SCI 55.6 (9.9) woSCI: 53.5 (9.4)	1	Acute	Healthy adults	Blood	CD56, CD4+ Tcells, CD8+ Tcells, B cells, NK cells, HLA-DR+ NK cells	NK cells, CD69+ NK cells, activation of NK cells, HLA-DR+ NK cells		Increase frequency and activated NK cells in peripheral blood of individuals with SCI in 24hrs.	Moderate (4)
Kliesch, 1996 United States [12]	49 Pp: 21 Tp: 28	32	No data	SCI: 25	[14-365]	Subacute-Intermediate-Chronic	Healthy adults	Blood	NK cell and Tcell function		NK cell and T cell function, CD56+	Tcell function peaks decrease at 2 weeks by recovers to normal values after 3 months. SCI without rehab has continued immune depression while those in rehab have their Tcell and NK cell function restored after 6 months.	Moderate (2)
Paczkowska, 2015 Poland [33]	20	25	SCI: 19 (95) woSCI: 25 (100)	SCI: 39.1 (19.2) woSCI: 35.6 (8.4)	[1-7]	Acute-Subacute	Healthy adults	Blood (Plasma)	CD34, CD133, RNA expression patterns of PB-derived nucleated cells	CD34+/CD133+/VEGFR2+ (day 1,3,7) CD34+/VEGFR2+ (day3)		The global gene expression changes revealed at the decreased expression of genes associated with regulation of immune system and immune response was observed in NCs in SCI vs woSCI.	Moderate (4)
Arevalo-Martin, 2018 Spain [35]	52 C:14 T: 23 L:2	16	SCI: 46 (88) woSCI: 13 (81)	SCI: 40.6 (15.9) woSCI: 45.0 (11.2)	0.08 (0.02)	Acute	Healthy adults	Blood (Serum)	Leukocytes, Neutrophil, Monocytes, Lymphocytes, Basophils, Eosinophils	Leukocytes (Complete), Neutrophils (Complete)	Lymphocytes (Complete)		Moderate (4)
Grassner, 2022 Austria/Spain [36]	81	26	SCI:72 (89) woSCI: 18 (69)	SCI: 42.1 (30.6) woSCI: 40.8 (21.9)	31 (9)	Intermediate	Healthy adults	Blood (Serum)	Leukocyte, Neutrophils, Lymphocytes, Monocytes, Eosinophils, Basophils	Leukocyte, Neutrophils, Eosinophils (only for those who developed infections)	Lymphocytes (only for those who developed infections)		Low (3)

										Neutrophil/Lympho cyte (for those who developed and did not develop infections)			
Wichmann, 2022 Denmark [13]	15 C:11 T:4	15	SCI: 11 (73) woSCI: 8 (53)	SCI: 50 (19) woSCI: 53.3 (20.1)	[0-199]	Acute-Subacute-Intermediate	Healthy adults	Blood (Plasma)	Neutrophils, Monocytes, Lymphocytes, Eosinophils, Mast Cells				Moderate (2)
Fraussen, 2022 [80]	18 C:12 T:5 L:1	18	SCI:16 (89) woSCI: 16 (89)	SCI: 56 (13) woSCI: 56 (15)	[0-126]	Acute-Subacute-Intermediate	Healthy adults	Blood (Plasma)	Monocytes, NK cells, Tcells, Bcells			Higher frequencies of circulating immune cell subsets (memory T and B memory T and B) in individuals with SCI compared to those woSCI. Changes were apparent in the B cell compartment, the decreased IgG+ and increased IgM+ B cell frequencies correlated with injury severity	Moderate (4)

SCI, spinal cord injury; woSCI, without SCI; CD, cluster differentiation; MHC, major histocompatibility complex; HLA, human leukocyte antigens; IL, interleukin; NK, natural killer; VEGF, vascular endothelial growth factor

Supplemental Table S5. Summary of the studies that measured or studied circulating immune cell populations during chronic phase among individuals with SCI compared to those without SCI.

Lead Author	SCI (n)	woSCI (n)	Male (%)	Age (sd) [range]	Year duration of injury (sd) [range]	SCI Population characteristics	Sample	Immunological markers used	Significantly Increased Markers	Significantly Decreased Markers	Other significant finding	Risk of bias (Quality of Evidence Rating)
Campagnolo, 1994 United States [37]	5 C:5	5	SCI: 4 (80) woSCI: 4 (80)	SCI: 36.2[20-69] woSCI: 35.1[19-68]	2.8 [0.6-10]	Healthy adults	Blood	Leukocytes, Neutrophil, Monocytes, Lymphocytes, Bcells, Tcells, Thelp:Tsupp, NK cells			There was a decrease in NK cell cytotoxic capacity among SCI but was not significantly different from woSCI.	Moderate (4)
Campagnolo, 1997 United States [38]	18 Pp:8 Tp:10	18	SCI: 11 (61)	SCI: [19-51]	[0.3-18.1]	Healthy adults	Blood	Neutrophil phagocytosis		Neutrophil phagocytosis (Tetraplegia)	Significantly impaired phagocytic ability in the tetraplegic group compared with their controls while the paraplegic group did not demonstrate these findings	Moderate (4)
Campagnolo, 2008 United States [39]	36	34	SCI: 33 (92) woSCI: no data	SCI: 37.1 (14.0) woSCI: 36.4 (13.0)	1.7 (3.6)	Healthy adults	Blood	Leukocytes, Neutrophil, Monocytes, Lymphocytes, Basophils, Eosinophils, Bcells, Tcells, Thelper, Tcytotoxic, NK cells	Tcells, Thelper, NK cells	NK cells, NK cell cytotoxicity	No difference in NK cytoxicity associated to the level of injury	Low (4)
Davies, 2007 Canada [40]	56 Pp: 25 Tp: 31 * only 10 Pp and 22 Tp were including as the others have complications	35	SCI: 42 (75) woSCI:18 (51)	SCI: 40.6 (11.9) woSCI: 35.1 (9.8)	10.7 (8.3)	Healthy adults	Blood (Serum)	Leukocytes, Neutrophil, Monocytes, Lymphocytes, Basophils, Eosinophils				Moderate (4)
Diaz, 2021 Spain [41]	55 C:24 T: 24 L:7	28	SCI: 37 (68) woSCI: 12 (43)	SCI: 26.9 (12.9) woSCI: 25.0 (2.9)	12.0 (9.22)	Healthy adults	Blood (Serum)	Monocytes, CD14, CD16			Significant decreased expression of TLR4 and TLR9+ in monocytes, CD14highCD16-, CD14high+CD16+ among SCI vs woSCI. Significant dcrease in percentage of monocytes that can phagocytosed *E.coli* among SCI vs woSCI	Low (4)

Formisano, 1998 Italy [42]	18 C:5 T: 11 L: 2	10	SCI: 17 (94) woSCI: 5 (50)	SCI: 32.7 (11.2) woSCI: 32.7	4.0 (6.11)	Healthy adults	Blood (Serum and Plasma)	CD3, CD19, CD4, CD8, CD16, HLA-DR, CD14, CD25		CD25	No significant difference in CD3, CD19, CD4, CD8, CD16, HLA-DR and CD14.	Moderate (4)
Gucluler, 2017 Turkey [43]	17	13	SCI: 14 (82) woSCI: 7 (54)	SCI: 35 (6.7) woSCI: 32.8 (7.5)	9.2 (6.3)	Healthy adults	Blood	Leukocytes, Neutrophil, Monocytes, Lymphocytes, Basophils, IP10-monocytes		IP10 producing monocytes	Significantly decreased TLR7 dependent IFNy and IP10 levels and TLR9 mediated APC function compared to woSCI. B cell and dendritic cells retain their functionality in response to IL6 and IFNa stimulation.	Moderate (4)
Hashizaki, 2018 Japan [3]	19 C:9 T:10	8	27 (100)	SCI: 38.6 (8.3) woSCI: 39.3 (2.8)	16.2 (11.5)	Healthy adults	Blood	Leukocytes, Monocytes			There was significant increase in leukocyte concentration in both individuals with SCI and woSCI in low body heat stress.	Moderate (2)
Kanyilmaz, 2013 Turkey [44]	34 Pp: 18 Tp: 16	28	SCI: 12 (35) woSCI: 9 (31)	SCI: 33.1 (12.1) woSCI: 34.5 (12.4)	1.4 (1.3)	Healthy adults	Blood (Plasma)	Neutrophil phagocytosis, mean intensity of fluorescence (MIF) of absorbed *E.coli*		Percentage of neutrophils in response to *E.coli*. Mean intensity of fluorescence (MIF) of absorbed *E.coli*	No significant difference among paraplegia vs tetraplegia or above T10 or below T10 SCI level with regards to neutrophil phagocytosis nor oxidative burst.	Moderate (4)
Hayes, 2002 Canada [45]	24 Pp: 4 Tp: 20	26	SCI: 19 (76) woSCI: 15 (58)	SCI: 36.8 (9.8) woSCI: 35.8 (8.2)	10.4 (8.1)	Healthy adults	Blood (Serum and Plasma)	Leukocytes	Leukocytes			Moderate (4)
Pan, 2005 Taiwan [46]	30 Pp: 14 Tp: 16	30	60 (100)	SCI: 38.5 (5.4)	median 8.5-9.5	Healthy adults	Blood	T cell reactivity, dendritic cell maturation		Phenotypic maturation (expression of CD80 and CD83 frequency) of dendritic cells	LPR abd CD69 expression of Tcells were similar in SCI vs woSCI. Dendritic cell maturation was significantly less in tetraplegia vs paraplegia. No significant correlation of dentritic cell maturity to duration of injury.	Moderate (3)
Monahan, 2015 United States [47]	23	11	No data	SCI: 56.2 (13.1) woSCI:50 .2 (9.95)	15.2 (10.4)	Healthy adults	Blood	Tcells and Tregs (CD3, CD4, CD8, HLA-DR)	activated CD4+ Tcells, CD4+ Tregs	CD3+ Tcells, CD3+ CD4 Tcells	HLA-DR expression (activated CD4+) increased among those with complete lesions and those with above T5 injury. The mean fluorescence intensity with CD4+ Tcells and CD8+ Tcells did not differ between SCI and woSCI	Moderate (4)
Lynch, 2002 New Zealand [77]	30 C: 20 T: 10	30	SCI: 26 (87) non SCI: 26 (87)	SCI: 44 [22-67]	>1	Healthy adults	Blood	Leukocytes	Leukocytes		No significant difference in Pneumovax response between SCI and woSCI and no difference with cervical vs thoracic SCI.	Moderate (3)

Pavlicek, 2017 Switzerland [48]	85	84	169 (100)	SCI: 55.63 (7.63) woSCI: 55.2 (8.06)	18.7 (15.6)	Healthy adults	Blood (Plasma)	Leukocyte, Monocyte, Lymphocytes, Neutrophils, Eosinophils, Basophils, CD4 and CD8 (Tn, Tscm, Tcm+tm, Tem, Tte)	Leukocyte, Neutrophils (older SCI), Lymphocytes (older SCI), Monocytes (older SCI), GM-CSF (young SCI), Tcm+tm% of CD4, Tn% of CD8 (older SCI),	Lymphocytes (young SCI), IL2 (young SCI), Tn% of CD4, Tte% of CD4 (young SCI), Tte% of CD8 (young SCI)	Significantly lower GM-CSF and telomere length in older SCI vs young SCI. Significantly higher G-CSF and CD4/8 in older SCI vs young SCI. Significantly higher TNFa among older SCI vs young SCI. Significantly higher monocytes among older SCI vs young SCI. Significantly increased Tem% of CD4 and Tte% of CD4 in older SCI vs young SCI. Significantly increased Tcm+tm% of CD8, Tem% of CD8 and Tte% of CD8 in older SCI vs young SCI. Significantly decreased IgG levels in older SCI vs young SCI.	Low (4)
Iversen, 2004 Norway [49]	12 Pp: 6 Tp: 6	6	SCI: 12 (100) woSCI: 6(100)	SCI: [24-42]	>5	Healthy adults	Blood,	Leukocytes, Neutrophil, Monocytes, Lymphocytes, Eosinophils			No significant difference in leukocytes, number of T and B cells between SCI vs woSCI and paraplegia vs tetraplegia.	Moderate (4)
Ueta, 2008 Japan [1]	7 Pp:7	6	13 (100)	SCI:34.3 (18.78) woSCI: 28.8 (18.86)	No data	Adults with regular physical training	Blood (Plasma)	Leukocytes, Lymphocytes, Monocytes, Neutrophils NK cells, NK cell actvity	Leukocytes, neutrophils (2hr after exercise) NK cell activity (pre-exercise)		No signifcant difference in NK cell count between SCI vs control pre-exercise. ACTH was significntly differen in exercise and immediately after among woSCI vs SCI.	Moderate (2)
Yamanaka, 2004 Japan [6]	8 C:8	6	14 (100)	SCI: 35.3 (9.62) woSCI: 34.3 (7.84)	[4.4-5.3]	Adults with regular physical training	Blood (Plasma)	Leukocytes, Lymphocytes, Monocytes, Neutrophils NK cells, NK cell actvity	Neutrophils (2hr after exercise)	NK cells (pre and post-exercise, 2hr recovery)	No signifcant difference in NK cell activity between SCI vs control pre-exercise. NK cell activity increased in woSCI while SCI was constant. ACTH and NK cells increase in exercise among woSCI but not with SCI. ACTH was significantly lower in SCI	Moderate (2)
Leicht, 2015 Japan [10]	7 C:7	10	17 (100)	SCI: 39 (12) woSCI: 41 (38)	9 (7)	Healthy adults	Blood (Plasma)	Leukocytes	Leukocytes			High (2)
Szymczak, 2021 Poland [2]	11	11	22 (100)	SCI: 34.2 (4.4) woSCI: 22.6 (2.4)		Athletes	Blood (Plasma)	Leukocytes, Lymphocytes, Monocytes, Granulocytes			Athletes with SCI had significantly lower change in leukocyte, lymphocyte and granulocytes concentration after exercise compared to athletes without SCI.	High (2)
Hsieh, 2009 Taiwan [50]	20 Pp:11 Tp: 9	20	No data	SCI: 44 (11.2) woSCI: no data	13.3 (6.2)	Healthy adults	Blood (Plasma)	Dendritic cells		Dendritic cell maturation	Nuclear factor (NF)-kB activity was signifantly lower in dendritic cells from tetraplegic vs paraplegics. Tat pretreatment increase the NF-kB but do not increase dendritic cell maturity.	Low (4)

Kouda, 2012<br>Japan [5]	8<br>C:8	8	16 (100)	SCI: 37.1 (9.3)<br>woSCI:32.4 (8.7)	[5.8-6.3]	Adults in regular training	Blood (Plasma)	Leukocytes, Monocytes, Lymphocytes, Neutrophils			No significant increase in leukocytes post-2hrs of exercise vs baseline while woSCI significantly increased. There was significant increase in neutrophils compared to baseline for both SCI and woSCI group.	Moderate (2)
Umemoto, 2011<br>Japan [4]	6<br>Pp:6	7	13 (100)	SCI: 30.7 (6.9)<br>woSCI: 29.4 (4.0)	No data	Adults with regular training	Blood (Plasma)	Leukocytes			Leukocytes increased after exercise in both SCI and woSCI group.	Moderate (2)
Pang, 2022<br>China [51]	23	21	SCI: 18 (78)<br>woSCI: 16 (76)	SCI: 35.9 (8.3)<br>woSCI: 37.1 (7.9)	[0-25]	Healthy Han Chinese adult	Blood (Plasma)	Leukocytes, Lymphocytes		Proportion of CD4+Tcells, CD4+CD8+, CD4+/CD8+	There is a disruption in the homeostasis of the CD4+Tcell subset in the SCI. There is no difference in CD3+ cells, CD8+Tcells, CD4-CD8-cells, NK cells, B cells and CIK cells	Moderate (4)
Iversen, 2002<br>Norway [87]	6<br>Tp:6	8	14(100)	SCI: 36 (8.1)<br>woSCI: 33 (4,.2)	11.5 (5.6)	Healthy adults	Blood (Plasma)	Leukocytes				Low (2)
Iversen, 2000<br>[82]	12<br>Tp:6<br>Pp:6	6	18 (100)	SCI: [36-49]<br>woSCI: [29-37]	[7-40]	Healthy adults	Blood	Lymphocyte cytoxicity		Lymphocyte cytoxicity		Moderate (4)
Shi, 2022<br>China [34]	108	52	SCI: 78 (72.2)<br>woSCI:35 (67.3)	SCI: 53.5 (6.7)<br>Non-SCI: 53.1(6.2)	No data	Healthy adults	Blood (Serum)	Leukocytes				Moderate (4)
Bernardi, 2019 [90]	25	25	50 (100)	SCI: 38.7 (2.5)<br>Non-SCI:38.7 (1.8)	No data	Para-Athletes	Blood (Serum)	Leukocytes, Neutrophils, Monocytes, Lymphocytes, Granulocytes		Lymphocytes		Moderate (4)

SCI, spinal cord injury; woSCI, without SCI; CD, cluster differentiation; TNF, tumor necrosis factor; GM-CSF, granulocyte-macrophage colony stimulating factor, NK, natural killer; MHC, major histocompatibility complex; HLA, human leukocyte antigens; IL, interleukin; IFN, interferon; ACTH, adrenocorticotropic hormone

Supplemental Table S6. Summary of the studies that measured circulating cytokines and chemokines during the acute, subacute and intermediate phase in individuals with SCI and compared to those without SCI.

Lead Author	SCI (n)	woSCI (n)	Male (%)	Age (sd) [range]	Day duration of injury (sd) [range]	Phase in SCI	SCI Population characteristics	Sample	Immunological markers used	Significantly Increased Markers vs woSCI	Significantly Decreased Markers vs woSCI	Other significant finding	Risk of bias (Quality of Evidence Rating)
Bank, 2015 United States [52]	18 C: 14 T: 4	18	SCI: 16 (89) woSCI: 15 (83)	SCI: 60 (21.21) woSCI: 58 (21.21)	[0-15]	Acute-Subacute	Healthy adults	Blood (Plasma)	b-FGF, CTACK, eotaxin, G-CSF, GM-CSF, GRO-a/CXCL1, HGF, IFN-a2, IFN-g, IL-1a, IL-1b, IL-1Ra, IL-2, IL-2Ra, IL-3, IL-4, IL-5, IL-6, IL-7, IL-8, IL-9, IL-10, IL-12p40, IL-12p70, IL-13, IL-15, IL-16, IL-17, IL-18, IP-10, LIF, MCP-1/CCL2, MCP-3/CCL7, MCSF, MIF, MIG/CXCL9, MIP1-a/ CCL3, MIP1-b/CCL4, b-NGF, PDGF-bb, RANTES/CCR1, SCF, SCGF-b, SDF1-a, TNF-a, TNF-b, TRAIL, and VEGF	MIF, IL6, IL9, IL16, IL18,CXCL1, MIP1-b/CCL4		No significant correlation with MIF to Glascow Coma Scale, Injurity Severity Score, injury level or mechanism of injury. The elevated chemokines have no significant correlation to sex, GCS, injury level, injury completeness, mechanism of injury, age, steroid treatment or LOS. Mean MIF levels correlated significantly with mean levels of IL6, IL9, IL16, IL18, GRO-a/CXCL1, MIP1-b/CCL4, HGF and SCGF-b	High (2)
Heller, 2021 Germany [31]	18	4	SCI: 15 (83) woSCI: 4 (100)	SCI: 49.7 (39.0) woSCI: 43 (median)	[0-90]	Acute-Subacute-Intermediate	Healthy adults	Blood (Plasma)	Monocyte (CD14, CD16, IL10, CXCL4), Lymphocyte transformation, Cytokines (CCL2, IL10, TGFb1, TGFb2)			Classical monocytes were lower among those with neurological remission vs to those who did not. Non-classical monocytes were lower among SCI vs control. Total lymphocytes had similar frequency compared to woSCI. Those with neurological remission had lymphocytes response recovered to elevated levels compared to non-remission group. Early elevated concentrations of CD14-/CD16+/IL10+/CXCR4int monocytes were elevated and related to higher odds of CNS regeneration.	Moderate (2)

Chen, 2020a China [59]	84	49	SCI: 56 (67) woSCI: 35(71)	SCI: 47.3 (5.0) woSCI:46.6 (5.0)	1	Acute	Healthy adults	Blood (Serum)	CCL21	CCL21		CCL21 is negatively correlated to the Montreal Cognitive Assessment.	Moderate (4)
Chen, 2020b China [60]	96	52	SCI: 60 (63) woSCI: 31 (60)	SCI: 50.2 (5.1) woSCI: 49.4 (5.2)	1	Acute	Healthy adults	Blood (Serum)	IL37	IL37		IL37 is negatively correlated with ASIA motor scores.	Moderate (4)
Hassanshahi, 2013 Iran [83]	78	70	SCI: 58 (74) woSCI: 38 (54)	SCI: 34.3 (2.3) woSCI: 38.4 (13.8)	[0-90]	Acute-Subacute-Intermediate	Healthy adults	Blood (Serum)	CXCL-1, CXCL-9, CXCL-10, CXCL-12	CXCL-1, CXCL-9, CXCL-10 (peaked at 7 days): CXCL-12 (peaked at 7 days and persisted for 28 days)		There was no correlation between age and chemokine levels. Expression on CXCL-12 were similar in both sexes and different age groups	Moderate (2)
Huang, 2013 United States [53]	27 C: 13 T: 13 L:1	18	SCI: 23 (85) WoSCI: 0 (0)	SCI: 40.8 (18.1)	[1-7]	Acute-Subacute	Healthy adults	Blood (Plasma)	Monocyte (CD14, CD16), CCL11, GM-CSF, IFN, IFN-y, IL-1Ra, IL-1b, IL-2, SIL-2RA, IL-4, IL-5, IL-6, IL-7, IL-8, IL-10, IL-12p40, IL-12p70, IL-13, IL-15, IL-17, CXCL10, MCP-1/CCL2, MIP-1°, MIP-1b, TNF-a, CXCL9	CD14+CD16+Monocyte		The total inflammatory monocyte population was not significantly different as M1 polarized or M2 polarized. Monocytes in SCI with AIS A/B with M1 dominance trended towards higher IL12p70 and IFNy while those with M2 dominance had higher IL10 and IL7.	Moderate (4)
Yong, 2018 China [54]	30 C: 30	20	SCI: 21(70) woSCI: 12 (60)	SCI: 54.9 (9.3) woSCI: 53.5 (9.3)	1	Acute	Healthy adults	Blood (Plasma)	IL1b, IL6, IL10, TNFa, TGFb1, IFNy	IL1b, IL6, TGFb1, IFNy		Significantly higher TGFb1 among those with complete lesion vs incomplete and no difference in IL1b and IL6.	Moderate (4)
Xu, 2019 China [32]	21 C: 21	20	SCI: 15 (71) woSCI: 11(55)	SCI 55.6 (9.9) woSCI: 53.5 (9.4)	1	Acute	Healthy adults	Blood	TGFb, IL17A, IL23p40	TGFb	IL17A, IL23p40		Moderate (4)
De Mello Rieder, 2018 Brazil [55]	52	36	SCI: 47 (90) woSCI: no data	SCI: 35.1 (14.5) woSCI:no data	[0-7]	Acute-Subacute	Healthy adults	Blood (Serum)	IL6	IL6		Significant difference was observed after 48hrs but no difference after 7 days vs woSCI.	Moderate (2)
Liu, 2005 China [56]	28 C:17 T:11	8	SCI: 19 (68) woSCI: no data	SCI: 36.2 [28-66] woSCI: 31.5 [22-61]	[0-3]	Acute-Subacute	Healthy adults	Blood (Serum)	MCP1	MCP1		MCP-1 was significantly lower in individuals with normal MRI finding.	Moderate (4)

Grassner, 2022 Austria/Spain [36]	81	26	SCI:72 (89) woSCI: 18 (69)	SCI: 42.1 (30.6) woSCI: 40.8 (21.9)	31 (9)	Intermediate	Healthy adults	Blood (Serum)	TGFb1, MCP1, CXCL1, CXCL10, CCL11		CCL11 (only for those who developed infections)<br><br>TGF1b (for those who developed and did not develop infections)	Other inflammatory markers such BNDF were significantly decreased for both groups while platelets were significantly increased compared to woSCI group.	Low (3)
Mi, 2019 China [62]	105 C:22 TL: 58 S:16	40	SCI: 62(59) woSCI: 25 (63)	SCI: 31.9 (7.58) woSCI: 31.1(7.23)	<1	Acute	Healthy adults	Blood (Serum)	TNFa, IL12, IL6	TNFa, IL12, IL6		Serum levels of TNFa, IL12, IL6 were negatively correlated to orthopedic scores (Japanese Orthopedic Association) improvement rate.	Moderate (4)
Wichmann, 2022 Denmark [13]	15 C:11 T:4	15	SCI: 11 (73) nonSCI: 8 (53)	SCI: 50 (19) nonSCI: 53.3 (20.1)	[0-199]	Acute-Subacute-Intermediate	Healthy adults	Blood (Plasma)	IL6, IL8, IFNy, TNFa, IL2, IL12p70, IL4, IL10, IL13	IL6, IL8, IL10	IFNy		Moderate (2)
Heller, 2017 Germany [15]	20	10	SCI: 16 (80) nonSCI: 5 (50)	SCI: 41.0 (19.6) nonSCI:43 (15)	[0-84]	Acute-Subacute-Intermediate	Healthy adults	Blood (Serum)	CCL2, CCL3, CCL4, CXCL5			Significant elevation difference in CCL2 and CCL4 among those with no neurological remission compared to those with neurological remission.	Moderate (2)

Supplemental Table S7. Summary of studies that measured circulating cytokines and chemokines during the chronic phase among individuals with SCI and compared to those without SCI.

**Lead Author, Location**	**SCI (n)**	**woSCI (n)**	**Male (%)**	**Age (sd) [range]**	**Year duration of injury (sd) [range]**	**SCI Population Characteristic**	**Sample**	**Immunological markers used**	**Significantly Increased Markers vs woSCI**	**Significantly Decreased Markers vs woSCI**	**Other significant finding**	**Risk of bias**
Wang, 2007 Taiwan [57]	62 Pp: 42 Tp:20	29	SCI: 62 (100) woSCI: 29 (100)	SCI: 40 (11) woSCI: 36(11)	12(7.0)	Healthy adults	Blood (Serum)	IL6, soluble CD40L, CRP	IL6, CRP		Chronic SCI with no evidence of infection have increased IL6 regardless on injury duration, injury levels compared woSCI.	Low (4)
Stein, 2013, United States [58]	22 C: 15 T: 7	19	SCI: 16(72) woSCI: 13(68)	SCI: 48 (14.1) woSCI: 41 (8.7)	12 (7.0)	Healthy adults	Blood (Plasma)	MIF, MIG/CXCL9, MCSF, IL3, SCGFb, IL1a, IL2RA, IL12p40, IL16, IL18, GROa, IFNa2, MCP3, SDF1a, bNGF, TNFb	MIF, MIG/CXCL9, MCSF, IL3			Moderate (4)
Matous-Souza, 2010 Brazil [80]	34	31	SCI: 34 (100) woSCI: 31(100)	SCI: 31.9 (7.58) woSCI: 31.1(7.23)	6.7 (4.66)	Healthy adults	Blood (Serum)	TNFreceptorI, TNFreceptorII, IL6, TGFb, Monocyte-stimulated (IL17, IFNy, IL6 and IL10), CRP	CRP, TNFreceptorI, TNFreceptorII, Monocyte stimulated IL6		SCI exhibited higher inflammatory markers compared to woSCI but no significant difference between tetraplegia vs paraplegia. Mononuclear cells were more activated in SCI.	Moderate (4)
Gucluler, 2017 Turkey [43]	17	13	SCI: 14 (82) woSCI: 7 (54)	SCI: 35 (6.7) woSCI: 32.8 (7.5)	9.2 (6.3)	Healthy adults	Blood	IL6, IL10, IFNy, IP10-monocytes		IP10 producing monocytes	Significantly decreased TLR7 dependent IFNy and IP10 levels and TLR9 mediated APC function compared to woSCI. B cell and dendritic cells retain their functionality in response to IL6 and IFNa stimulation.	Moderate (4)
Hayes, 2002 Canada [45]	24 Pp: 4 Tp: 20	26	SCI: 19 (76) woSCI: 15 (58)	SCI: 36.8 (9.8) woSCI: 35.8 (8.2)	10.4 (8.1)	Healthy adults	Blood (Serum)	IL2, IL4, IL10, TNFa,	IL2, IL4			Moderate (4)
Pavlicek, 2017 Switzerland [48]	85	84	SCI: 85 (100) woSCI: 84 (100)	SCI: 55.63 (7.63) woSCI: 55.2 (8.06)	18.7 (15.6)	Healthy adults	Blood (Plasma)	IL2, IL4, IL10, G-CSF, GM-CSF, MCP-1, CD4/8, IL6, TNFa, CRP		TNFa, IL2 (young SCI), IL4, G-CSF, MCP-1 (older SCI),	Significantly lower GM-CSF and telomere length in older SCI vs young SCI. Significantly higher G-CSF and CD4/8 in older SCI vs young SCI. Significantly higher TNFa among older SCI vs young SCI.	Low (4)
Diaz, 2021 Spain [41]	55 C:24 T: 24 L:7	28	SCI: 37 (68) woSCI: 12 (43)	SCI: 26.9 (12.9) woSCI: 25.0 (2.9)	12.0 (9.22)	Healthy adults	Blood (Serum)	IL6, IL10, IL1b, TNFa	IL6, TNFa			Low (4)

Baria, 2007 United States [83]	10	10	SCI: 7(70) woSCI:7 (70)	SCI: 39.9 (9.7) woSCI: 39.8 (9.6)	>1yr	Physically active	Blood	TGFb	TGFb			Moderate (4)
Rogeri, 2005 Brazil [65]	7	11	SCI: no data woSCI:11 (100)	SCI: no data woSCI:21.4(6.0)	No data	Healthy adults	Blood (Plasma)	TNFa, IL1a		IL1a, TNFa	Significantly lower plasma glutamine among SCI vs woSCI.	Moderate (4)
Leicht, 2015 Japan [10]	7 C:7	10	17 (100)	SCI: 39 (12)<br><br>WoSCI: 41 (38)	9 (7)	Healthy adults	Blood (Serum)	IL6, IL1RA, IL8, TNFa,	IL6, IL8, IL1RA,			High (2)
Hashizaki, 2018 Japan [3]	19 C:9 T:10	8	27 (100)	SCI: 38.6 (8.3) woSCI: 39.3 (2.8)	16.2 (11.5)	Healthy adults	Blood (Serum)	TNFa, CRP, IL6	IL6		There was significant increase in IL6 concentration in both individuals with SCI and woSCI in low body heat stress. There was significantly higher in increase in IL6 in individuals without SCI compared to those with cervical level SCI.	Moderate (2)
Frost, 2005 United States [66]	34 (26 without pressure ulcers)	10	44(100)	SCI: 43 (11.9) woSCI: 30.1 (2.44)	10.8 (8.2)	Healthy adults	Blood (Serum)	CRP, TNFa, IL6	CRP		No significant difference in TNFa and IL6 and no difference in CRP, TNFa and IL6 between paraplegia vs tetraplegia.	Low (4)
Radulovic, 2015 United States [67]	12	10	22 (100)	SCI: 48 (11) woSCI: 48 (10)	21 (14)	Healthy adults	Blood (Serum)	TNFa, IL6			No significant difference in the tested cytokines.	¨Moderate (4)
Liang, 2008 United States [68]	129	129	258(100)	SCI: 37 (10.5) woSCI: 36.7 (11.2)	Atleast 1 year	Healthy adults	Blood (Seum)	CRP	CRP		Individuals with complete injury had significantly higher CRP compared to those with incomplete injury. Complete injury, age, smoking, weight, and waist circumference were significant predictors for elevated CRP.	Low (4)

Wang, 2009 [69]	110	62	172 (100)	SCI: 39.6 (10.2)<br>woSCI: 40.6 (8.8)	12.5 (6.9)	Healthy adults	Blood (Serum)	hs-CRP, IL1b	hs-CRP		No difference in hs-CRP concentration between SCI group with above T6 lesion and below T7 lesion. There was significant difference in IL1b between those with above T6 (higher) vs below T7.	Low (4)
Umemoto, 2011<br>Japan [4]	6<br>Pp:6	7	13 (100)	SCI: 30.7 (6.9)<br>woSCI: 29.4 (4.0)	No data	Adults with reular training	Blood (Plasma)	CRP, TNFa			IL6 significantly increased after exercise for both SCI and woSCI groups while TNFa and hs-CRP did not change.	Moderate (2)
Brewster, 2020<br>Canada [70]	8<br>C:8	10	SCI: 7(87)<br>woSCI: 7 (70)	[27-64]	26 (14.1)	Healthy adults	Blood (plasma)	IL6, TNFa			The microvesicles isolated from SCI adults reduced eNOS activation, NO production and t-PA expression. Diminished eNOS activity, NO bioavailaility and t-PA production leads to higher susceptible to atherosclerotic and thrombogenic processes.	Moderate (4)
Diaz, 2021<br>Spain [41]	55<br>C:24<br>T: 24<br>L:7	28	SCI: 37 (68)<br>woSCI: 12 (43)	SCI: 26.9 (12.9)<br>woSCI: 25.0 (2.9)	12.0 (9.22)	Healthy adults	Blood (Serum)	IIL6, TNFa, IL10, IL1b	IL6, TNFa			Moderate (4)
Kouda, 2012<br>Japan [5]	8<br>C:8	8	16 (100)	SCI: 37.1 (9.3)<br>woSCI:32.4 (8.7)	[5.8-6.3]	Adults in regular training	Blood (Plasma)	IL6	IL6			Moderate (2)
Iversen, 2002<br>Norway [87]	6<br>Tp:6	8	14(100)	SCI: 36 (8.1)<br>woSCI: 33 (4,.2)	11.5 (5.6)	Healthy adults	Blood (Plasma)	CRP				Moderate (2)
Shi, 2022<br>China [34]	108	52	SCI: 78 (72.2)<br>woSCI:35 (67.3)	SCI: 53.5 (6.7)<br>Non-SCI: 53.1(6.2)	>24	Healthy adults	Bloood (Serum)	CRP			There were significantly higher levels of Nogo-A, a neurite outgrowth inhibitory factor, among individuals with SCI compared to individuals witout SCI.	Moderate (4)

Schreiber, 2017 Brazil [89]	22	11	33 (100)	SCI: 34.3 (8.6) woSCI: 28.9 (4.5)	7.1 (4.1)	Healthy adults	Blood (Plasma)	CRP				Moderate (4)
Edwards, 2008 Canada [86]	15	16	SCI: 12 (75) woSCI: 12 (80)	SCI: 38.1 (8.4) woSCI: 39.8 (7.4)	>1	Healthy adults	Blood (Serum)	CRP				Moderate (4)
Invernizzi, 2015 Italy [88]	28	15	SCI: 23 (82) woSCI: 5(33)	SCI: 40.5 (7.1) woSCI: 28.4 (4.1)	7.56 (4.43)	Healthy adults	Blood (Serum)	CRP	CRP			Moderate (4)
La Favor, 2011 United States [85]	14	13	SCI: 8 (57) woSCI: 9 (69)	SCI: 34.8 (11.5) woSCI: 34.0 (10.5)	>2	Healthy adults	Blood (Serum)	hsCRP	hsCRP			Moderate (4)
Liebermann, 2014 United States [84]	100	100	SCI: 78 (78) woSCI: 78 (78)	SCI: 44.8 (4.4) woSCI:45.3 (5.1)	15.1 (9.6)	Healthy adults	Blood (Serum)	hsCRP				Moderate (4)
Segal, 1993 United States [63]	42	18	60 (100)	SCI: 51.7 (12.7) woSCI:42.3 (15.3)	21.4 (13.6)	Healthy adults	Blood (Serum)	IL1b, IL2R			IL2R was significantly higher in quadriplegics	Moderate (4)
Bernardi, 2019 [90]	25	25	50 (100)	SCI: 38.7 (2.5) woSCI:38.7 (1.8)	No data	Para-Athletes	Blood (Serum)	CRP				Moderate (4)

Supplemental Table S8**.** Summary of studies that compared immunoglobulins, serum antigens to diseases or circulating antibodies among individuals with SCI compared to those without SCI.

**Author, Year Location**	**SCI (n)**	**woSCI (n)**	**Male (%)**	**Age (sd) [range]**	**Year duration of injury (sd) [range]**	**SCI Population characteristics**	**Sample**	**Immunological markers used**	**Significantly Increased Markers vs woSCI**	**Significantly Decreased Markers vs woSCI**	**Other significant finding**	**Risk of bias (Quality of Evidence Rating)**
Palmers, 2016 United States [71]	51 Pp: 26 Tp:25	49	SCI: 42 (82) woSCI: 32 (65)	SCI: 57 (16) woSCI: 55(17)	[0-21]	Healthy adults	Blood	Immunoreactivity to serological antigen selection for antibody profiling of SCI plasma			There was 51% of patients showed reactivity to at least 1 of 9 validated targets. Both traumatic and pathologic SCI has reactivity to the targets. SCI patients showed reactivity at 0 and 3 weeks after injury or surgery.	Moderate (2)
Arevalo-Martin, 2018 Spain [35]	52 C:14 T: 23 L:2	16	SCI: 46 (88) woSCI: 13 (81)	SCI: 40.6 (15.9) woSCI: 45.0 (11.2)	0.08 (0.02)	Healthy adults	Blood	IgG, IgM,	IgG (Cervical AIS A)		No significant difference in IgM between SCI vs woSCI. Autoantibodies targeting nervous system and systemic self-antigens related to changes in SCI are increasing at subacute phase of an SCI and are present in healthy SCI.	Moderate (4)
Hayes, 2002 Canada [45]	24 Pp: 4 T: 20	26	SCI: 19 (76) woSCI: 15 (58)	SCI: 36.8 (9.8) woSCI: 35.8 (8.2)	10.4 (8.1)	Healthy adults	Blood	anti-GM1 IgG, anti-GM1 IgM	anti-GM1 IgM			Moderate (4)
Pavlicek, 2017 Switzerland [48]	85	84	SCI: 85 (100) woSCI: 84 (100)	SCI: 55.63 (7.63) woSCI: 55.2 (8.06)	18.7 (15.6)	Healthy adults	Blood	IgG, CMV			Significantly decreased IgG levels in older SCI vs young SCI. Significant increase of CMV prevalence in older woSCI and SCI.	Low (4)
Shnawa, 2020 United States [72]	29 C: 17 T:10 L:2	25	SCI: 24 (83) woSCI: 16(64)	SCI: 53.9 (15.1) woSCI: 47.6(11.5)	15.3 (12.4)	Healthy adults	Blood	IgA, IgM, IgG1, IgG2, IgG3, IgG4	IgA, IgG2		The elevated IgA was significant regardless of, age, level of injury, time of injury vs woSCI but was significantly elevated only to AIS AB vs woSCI. The elevated IgG2 was only significant in neurological lesions at or above T5.	Low (4)
Chinigo, 1996 United States [73]	80 Pp: 41 Tp: 39	77	No data	SCI: 51.2 (12.7) woSCI: 68 (18)	17.7 (12.7)	Healthy adults	Blood	HBsAg, anti-HBsAb, anti-HBcAb			No significant difference in the prevalence of hepatitis B among SCI vs woSCI	Moderate (4)

Everall, 1974 United Kingdom [74]	79	297	SCI: 56(73) woSCI:137 (46)	No data	No data	Healthy adults	Blood	Agglutinin and precipitin titers to *Candida albicans*			Increase agglutinin titers among SCI compared to woSCI.	High (4)
Trautner, 2004 United States [75]	40 Pp: 25 Tp: 15	40	No data	SCI:41 woSCI:54	No data	Healthy adults	Blood	Serum antibody response to influenza vaccine			Serum antibody response were similar to control group. Vaccine response did not differ with level of lesion. Age 65 and older had significant reduction in antibody response compared to younger participants.	Moderate (3)
Rosman, 1998 United States [76]	78	93	SCI: 78 (100) woSCI: 92 (98)	SCI: 51.4 (15.8) woSCI: 68.7 (8.7)	> 3mos	Healthy adults	Blood	HBsAg, anti-HBsAb, anti-HBcAb			No significant difference between SCI group vs non-alcoholic general medicine group indicating that having SCI is not a independent risk factor in developing Hepatitis B or C.	Moderate (4)
Iversen, 2000 Norway [82]	12 Tp:6 Pp:6	6	18 (100)	SCI: [36-49] woSCI: [29-37]	[7-40]	Healthy adults	Blood	IgG		IgG		Moderate (4)

Supplemental Table S9. Summary of studies that compared immune markers in the seminal fluid among individuals with SCI compared to those without SCI.

Author, Year Location	SCI (n)	woSCI (n)	Age-years (sd) [range]	Duration of injury-years(sd) [range]	SCI Population characteristics	Immunological markers used	Significantly Increased Markers vs woSCI	Significantly Decreased Markers vs woSCI	Other significant finding	Risk of bias (Quality of Evidence Rating)
Aird, 1999 United Kingdom [16]	9	7	SCI: 34.7 [28-43] woSCI: 27.8 [19-36]	13.6 (6-43)	Healthy adults	CD3, CD4, CD8, CD14, CD16, CD22, CD45, CD69, Class II MHC, IgG	Retrograde:Proportion of leukocytes in SCI, CD3, CD16, CD22	-	The proportion of leukocytes in semen samples from men with SCIs was not significantly affected by the level, extent, or duration of injury or by the method of bladder management used.<br>Significant decrease in sperm motility.	Moderate (4)
Basu, 2002 United States [17]	12	8	SCI: 30.2 (4.2) woSCI 30.3 (5.1)	6.2 (3.8)	Healthy adults	Leukocyte CD45+		-	Significant decrease in mean sperm viability and sperm motility among males with SCI. Sperm concentration is similar.<br>Noted increase in leukocyte counts among SCI and increase in lymphocyte, monocyte and granulocyte. The majority of lymphocytes were T cells with predominance of CD4+.	Low (4)
Brackett, 2008 United States [18]	10	12	SCI: 32.9 (7.3)<br><br>woSCI: 33.8 (7.3)	7.9 (6.3)	Healthy adults	Leukocyte	Leukocyte	-	Significant decrease in sperm motility among males with SCI. Sperm concentration is similar.	Low (4)
da Silva, 2010 Brazil [20]	14	14	SCI: 32.6 (5.5) woSCI: 33.4 (9.1)	10.5 [1.8-28]	Healthy adults	Neutrophils	Neutrophils	-	Individuals with SCI and control groups were compared, no statistically significant differences were found with respect to concentration or total sperm count; however, the SCI group presented significantly lower ejaculate volume, decreased sperm morphology, and an increase in the round cell and neutrophils counts.	Low (4)
da Silva, 2013, Brazil [21]	12	10	SCI: 30.35 (7.30) woSCI: 35.5 (5.27)	8.3 (5.1)	Healthy adults	Neutrophils	Neutrophils	-	SCI is responsible for alterations in seminal plasmaprotein profile leading to a deviation fromhomeostasis; proteins reported in bothPVSand EEJ groups correlate with the pathophysiologyofSCI-related infertility.	Moderate (4)
Padron, 1997, United States [22]	24	19	SCI: 35 (1.5) woSCI: 33 (1.5)	4.7 (2.3)	Healthy adults	Leukocytes	Leukocytes	-	Levels of reactive oxygen species was significantly higher among males with SCI comapared to woSCI. Likewise, males with SCI had significantly lower morphologically normal spermatozoa.	Moderate (4)
Salsabili, 2006 Iran [23]	89	49	SCI: 34 (3.7) woSCI: 36 (4.8)	[11-18]	Healthy adults	Leukocyte	Leukocyte		Significantly decreased sperm count, percent motility, percent viability, percent normal form, percent sperm motility among SCI males.	Low (4)

Trabulsi, 2002 United States [24]	17	13	No data	No data	Healthy adults	Total leukocyte, Total WBC, CD4+ Tcell, CD8+ Tcell, B cell, Neutrophil, Macrophage	Total leukocyte, Neutrophil, Macrophage	-	Noted increase B cell, CD8+ Tcell, CD4+ Tcell, Total Tcell	Moderate (4)
Basu, 2004 United States [25]	31	12	SCI: 32 (11.7) woSCI 30.7 (12.5)	8.1(5.0)	Healthy adults	Th1 cytokines :IL1b, IL2, IL12, TNFa, TNFb, IFNy Th2 cytokines : IL4, IL6, IL10, TGFb1	IL1b, IL12, TNFa, IFNy IL6	TGFb1	Significant decrease in sperm count and sperm motility among males with SCI. IL1b, IL6, TNFa were not detected in serum of males with SCI	Low (4)
Brackett, 2007 United States [19]	11	5	SCI: 31.9 (7.6) woSCI:30.7 (8.0)	9 (6.6)	Healthy adults	TNFa, IL6, IL1b	TNFa, IL6, IL1b	-	The degree of improvement in sperm motility seemed to be related to the cytokine concentrations or starting sperm motility in the semen of SCI subjects. Neutralization of cytokine activity improved sperm motility.	Moderate (4)
Zhang, 2013 United States [26]	28	28	SCI: 35.0 (8.5) woSCI: 31.0 (8.5)	>1	Healthy adults	IL1b, IL18	IL1b, IL18	-	The median sperm concentration in the SCI group was not statistically significant compared with the control group. In contrast, the median sperm motility in the SCI group was significantly lower than the control group. The semen of most men with SCI contains normal sperm concentrations but abnormally low sperm motility. Elevated concentrations of inflammatory cytokines in the semen are detected in this unusual semen profile.	Low (4)

Supplemental Table S10. Summary of studies that compared the immune markers in the urine and the urothelium among individuals with SCI compared to those without SCI.

Author, Year Location	SCI (n)	woSCI (n)	Male (%)	Age-years (sd) [range]	Duration of injury-years(sd) [range]	SCI Population characteristics	Sample	Immunological markers used	Significantly Increased Markers vs woSCI	Significantly Decreased Markers vs woSCI	Other significant finding	Risk of bias (Quality of Evidence Rating)
Chen, 2016b Taiwan [7]	20 C:7 T: 11 L:2	10	SCI: 11 (55) woSCI: 0 (0)	SCI: 48.2 (18.4) woSCI: 52.40 (10.51)	chronic SCI	Healthy adults	Urothelium biopsy	Mast cell	-	-	Urothelial E-cadherin, ZO1 significantly lower in SCI vs woSCI but with higer TUNEL. BoNT-A injection improves E-cadherin and ZO1 in SCI to similar levels to non-SCI after 6mos while TUNEL remain significantly higher.	Moderate (2)
Chen, 2016a Taiwan [8]	26 C:12 T:14	10	SCI: 17(65) woSCI: 0(0)	SCI: 42.7(13.1) woSCI: 51.4 (12.1)	chronic SCI	Healthy adults	Urothelium biopsy	Mast cell	Mast cell	-	Urothelial E-cadherin, ZO1 significantly lower in SCI vs woSCI but with higer TUNEL. After 6 months of BoNT-A injection, ZO1 in SCI is significantly lower vs non-SCI while E-cadherin, mast cell and TUNEL remain significantly higher.	Moderate (2)
Jiang, 2015 Taiwan [9]	34 C:15 T: 19	10	SCI: 22 (65) woSCI: 0 (0)	SCI: 37.9 (11.8) woSCI: 50.5 (9.6)	9.3 (8.4)	Neurogenic detrusor overactivity	Urothelium biopsy	Mast cell	Mast cell	-	Significantly decreased E-cadherin, ZO1 and significantly increase apoptotic cell in the urothelium among SCI vs woSCI. The expression E-cadherin significantly negatively correlated with mast cell numbers. There was no difference in level of injury.	Moderate (2)
Vaidyanathan, 2000 United Kingdom [78]	46	10	No data	No data	No data	Individuals with neuropathic bladder	Urothelium biopsy	Immunostaining for sIgA in the vesical urothelium	-	-	All controls have strong cytoplasmic immunostaining for sIgA in the superficial layer of the transitional epithelium. sIgA immunoreactivity was absent in squamous metaplasia. In 37 that have transitional epithelium, 18 had moderate to intense immunostaining and 19 had absent to faint immunostaining.	High (4)
Pavlicek, 2017 Switzerland [48]	85	84	SCI: 85 (100) woSCI: 84 (100)	SCI: 55.63 (7.63) woSCI: 55.2 (8.06)	18.7 (15.6)	Healthy adults	Urine	Urine IgA	Urine IgA		-	Low (4)
Pang, 2022 China [51]	23	21	SCI: 18 (78) woSCI: 16 (76)	SCI: 35.9 (8.3) woSCI: 37.1 (7.9)	[1-300] mos	Healthy Han Chinese adult	Blood, Urine	Leukocytes, Lymphocytes, Urinary Leukocytes	-	-	Increased urinary leukocytes and urinary bacteria among individuals with SCI compared to woSCI.	Moderate (4)
Gomez, 1983 Spain [79]	28 C:13 T:11 L:4	10	No data	SCI: 38.5 (16.5)	Acute period	Healthy adults	Urine	IgG, IgA	IgA (C), IgG (C)		The increased in IgG and IgA in individuals with cervical lesions were associated with frequency of detected asymptomatic bacteriuria	Moderate (4)

Supplemental Table S11. Summary of studies that examined the immune markers in the bone marrow and saliva among individuals with SCI and compared them to those without SCI.

Author, Year Location	SCI (n)	woSCI (n)	Male (%)	Age-years (sd) [range]	Duration of injury-years(sd) [range]	SCI Population characteristics	Sample	Immunological markers used	Significantly Increased Markers vs woSCI	Significantly Decreased Markers vs woSCI	Other significant finding	Risk of bias (Quality of Evidence Rating)
Chernykh, 2006 Russia [81]	16 C: 9 T: 7	10	SCI: 12 (75)	SCI: [18-56]	2.2 [0.7-5]	Healthy adults	Bone marrow aspirate	Mononuclear cells, $CD34^+$(%, counts, G_0/G_1 %, S/G_2M%, apoptosis%), $CD34^+CD38^-$(%), Percentages of lymphocyte subpopulations	Mononuclear cells, $CD34^+$ (Thoracic), $CD34^+CD38^-$,$CD34^+$ (S/G_2M%), $CD34^+$apoptosis (Thoracic)	$CD34^+$(G_0/G_1%)	No significant difference in the lymphocyte subpopulation compared to woSCI.	Moderate (4)
Iversen, 2000 Norway [82]	12 Tp:6 Pp:6	6	18 (100)	SCI: [35-54] woSCI: [29-37]	[7-40]	Healthy adults	Bone marrow aspirate	NK cells, T cells, B cell, mononuclear cells, colony formation of progenitor cells.		NK cell cytotoxic capacity. Progenitor cells from the bone marrow below the site of injury had significantly reduced ability to form all types of colonies from the bone marrow. Progenitor cells collected below the site of injury had reduced ability to form dendritic cell colonies.		Moderate (4)
Iversen, 2004 Norway [49]	12 Pp: 6 Tp:6	6	SCI: 12 (100) woSCI: 6(100)	SCI: [24-42]	>5	Healthy adults	Bone marrow aspirate	CD3, CD19, CD34, CD45, CD16/56, Leukocytes, Neutrophil, Monocytes, Lymphocytes, Eosinophils			No significant difference in leukocytes, number of T and B cells between SCI vs woSCI and paraplegia vs tetraplegia. No apparent difference in any bone marrow cell type among SCI vs woSCI.	Moderate (4)
Leicht, 2011 United Kingdom [11]	15 Pp: 7 Tp: 8	8	SCI: 15 (100) woSCI: 8(100)	SCI:28.6 (4.5) woSCI:24 (6.2)	13.3 (7.2)	Athletes	Saliva	Salivary IgA concentration			sIgA secretion rate after exercise do not differ when comparing SCI vs woSCI or between paraplegia vs tetraplegia. The sIgA significantly increased after 60 min of both CL and IM exercise and sustained after 30min post exercise.	High (2)

Supplemental Table S12. Summary of studies that investigate the circulating immune cells' functional activity and ability to mature, phagocytose or produce oxidative burst among individuals with SCI and compared to those without SCI.

Lead Author	SCI (n)	woSCI (n)	Male (%)	Age (sd) [range]	Year duration of injury [range]	SCI Phase	SCI Population characteristics	Sample	Immunological markers used	Significantly Increased Markers	Significantly Decreased Markers	Other significant finding	Risk of bias (Quality of Evidence Rating)
Bao, 2011, Bao, 2009 Canada [27,28]	9 C:3 T:3 L:3	16	SCI: 7 (78) woSCI : 12 (75)	SCI: 44 (20.4) woSCI: 39.9 (13.1)	[0-14] days	Acute-Subacute	Healthy adults	Blood (Plasma)	Leukocytes, neutrophil, monocytes, lymphocytes	Neutrophils, monocytes		Oxidative capacity of neutrophils and monocytes was significantly increased compared to trauma controls. Lymphocytes had no evidence of oxidative burst activity. Difference in beta-integrins expression between trauma control and SCI after 24-48hrs injury.	Moderate (4,2)
Kliesch, 1996 United States [12]	49 Pp: 21 Tp: 28	32	No data	SCI: 25	[14-365] days	Subacute-Intermediate-Chronic	Healthy adults	Blood	NK cell and Tcell function,		NK cell and T cell function, CD56+	Tcell function peaks decrease at 2 weeks by recovers to normal values after 3 months. SCI without rehab has continued immune depression while those in rehab have their Tcell and NK cell function restored after 6 months.	Moderate (2)
Campagnolo, 1994 United States [37]	5 C:5	5	SCI: 4 (80) woSCI : 4 (80)	SCI: 36.2[20-69] woSCI: 35.1[19-68]	2.8 [0.6-10]	Chronic	Healthy adults	Blood	Leukocytes, Neutrophil, Monocytes, Lymphocytes, Bcells, Tcells, Thelp:Tsupp, NK cells			There was a decrease in NK cell cytotoxic capacity among SCI but was not significantly different from woSCI.	Moderate (4)
Campagnolo, 1997 United States [38]	18 Pp:8 Tp:10	18	SCI: 11 (61)	SCI: [19-51]	[0.3-18.1]	Chronic	Healthy adults	Blood	Neutrophil phagocytosis		Neutrophil phagocytosis (Tetraplegia)	Significantly impaired phagocytic ability in the tetraplegic group compared with their controls while the paraplegic group did not demonstrate these findings	Moderate (4)
Campagnolo, 2008 United States [39]	36	34	SCI: 33 (92) woSCI : no data	SCI: 37.1 (14.0) woSCI: 36.4 (13.0)	1.7 (3.6)	Chronic	Healthy adults	Blood	Leukocytes, Neutrophil, Monocytes, Lymphocytes, Basophils, Eosinophils, Bcells, Tcells, Thelper, Tcytotoxic, NK cells	Tcells, Thelper,	NK cells, NK cell cytotoxicity	No difference in NK cytoxicity associated to the level of injury	Low (4)

Formisano, 1998 Italy [42]	18 C:5 T: 11 L: 2	10	SCI: 17 (94) woSCI : 5 (50)	SCI: 32.7 (11.2) woSCI: 32.7	4.0 (6.11)	Chronic	Healthy adults	Blood	CD3, CD19, CD4, CD8, CD16, HLA-DR, CD14, CD25		CD25	No significant difference in CD3, CD19, CD4, CD8, CD16, HLA-DR and CD14.	Moderate (4)
Kanyilmaz, 2013 Turkey [44]	34 Pp: 18 Tp: 16	28	SCI: 12 (35) woSCI : 9 (31)	SCI: 33.1 (12.1) woSCI: 34.5 (12.4)	1.4 (1.3)	Chronic	Healthy adults	Blood (Plasma)	Neutrophil phagocytosis, mean intensity of fluorescence (MIF) of absorbed E.coli		Percentage of neutrophils in response to E.coli. Mean intensity of fluorescence (MIF) of absorbed E.coli	No significant difference among paraplegia vs tetraplegia or above T10 or below T10 SCI level with regards to neutrophil phagocytosis nor oxidative burst.	Moderate (4)
Pan, 2005 Taiwan [46]	30 Pp: 14 Tp: 16	30	60 (100)	SCI: 38.5 (5.4)	median 8.5-9.5	Chronic	Healthy adults	Blood	T cell reactivity, dendritic cell maturation		Phenotypic maturation (expression of CD80 and CD83 frequency) of dendritic cells	LPR abd CD69 expression of Tcells were similar in SCI vs woSCI. Dendritic cell maturation was significantly less in tetraplegia vs paraplegia. No significant correlation of dentritic cell maturity to duration of injury.	Moderate (3)
Yamanaka, 2004 Japan [6]	8 C:8	6	14 (100)	SCI: 35.3 (9.62) woSCI: 34.3 (7.84)	[4.4-5.3]	Chronic	Adults with regular physical training	Blood (Plasma)	Leukocytes, Lymphocytes, Monocytes, Neutrophils NK cells, NK cell actvity	Neutrophils (2hr after exercise)	NK cells (pre and post-exercise, 2hr recovery)	No signifcant difference in NK cell activity between SCI vs control pre-exercise. NK cell activity increased in woSCI while SCI was constant. ACTH and NK cells increase in exercise among woSCI but not with SCI. ACTH was significantly lower in SCI	Moderate(2)
Ueta, 2008 Japan [1]	7 Pp:7	6	13 (100)	SCI:34.3 (18.78) woSCI: 28.8 (18.86)	No data	Chronic	Adults with regular physical training	Blood (Plasma)	Leukocytes, Lymphocytes, Monocytes, Neutrophils NK cells, NK cell actvity	Leukocytes, neutrophils (2hr after exercise) NK cell activity (pre-exercise)		No signifcant difference in NK cell count between SCI vs control pre-exercise. ACTH was significntly different in exercise and immediately after among woSCI vs SCI.	Moderate (2)
Hsieh, 2009 Taiwan [50]	20 Pp:11 Tp: 9	20	No data	SCI: 44 (11.2) woSCI: no data	13.3 (6.2)	Chronic	Healthy adults	Blood (Plasma)	Dendritic cells		Dendritic cell maturation	Nuclear factor (NF)-kB activity was signifantly lower in dendritic cells from tetraplegic vs paraplegics. Tat pretreatment increase the NF-kB but do not increase dendritic cell maturity.	Low (4)

Iversen, 2000 Norway [82]	12 Pp:6 Tp:6	6	18 (100)	SCI: [35-54] woSCI: [29-37]	[7-40]	Chronic	Healthy adults	Blood	Lymphocyte cytoxicity		Lymphocyte cytoxicity		Moderate (4)
Diaz, 2021 Spain [41]	55 C:24 T: 24 L:7	28	SCI: 37 (68) woSCI: 12 (43)	SCI: 26.9 (12.9) woSCI: 25.0 (2.9)	12.0 (9.22)	Chronic	Healthy adults	Blood (Serum)	Monocytes, CD14, CD16			Significant decreased expression of TLR4 and TLR9+ in monocytes, CD14highCD16-, CD14high+CD16+ among SCI vs woSCI. Significant decrease in percentage of monocytes that can phagocytosed *E.coli* among SCI vs woSCI	Low (4)

Supplemental Table S13. Summary of the results of the subgroup analysis performed for the circulating immune markers that were included in the meta-analysis of studies comparing individuals with SCI vs those without SCI.

Circulating Immune Marker	Subgroups	Pooled Studies (N)	SCI (N)	woSCI (N)	WMD	95% CI	I^2 (%)	$\chi^2_{subgroup}$
**Leukocytes**	Phase							**<0.0001**
	Acute_Subacute	5	188	148	**2.92**	1.68, 4.16	74	
	Chronic	18	428	356	0.17	-0.28, 0.62	63	
	Completeness of Injury							**0.0361**
	Complete	7	80	70	0.03	-0.49, 0.56	0	
	Mixed	16	536	434	**1.03**	0.26, 1.81	88	
	Sympathetic Impairment							**0.0351**
	Above T6	9	95	76	0.03	-0.55, 0.61	15	
	Mixed	14	521	428	**1.09**	0.29, 1.87	89	
	Presence of Females							**0.0155**
	All Males	11	194	177	0.02	-0.55, 0.58	53	
	With Females	12	422	327	**1.39**	0.43, 2.35	88	
	Age Groups							**0.0002**
	With Seniors	12	462	368	**1.56**	0.68, 2.44	87	
	Adults	11	154	136	-0.26	-0.66, 0.13	5	
	Location							0.6362
	Europe and the Americas	14	299	284	0.64	-0.14, 1.42	81	
	Asia and the Pacific	9	317	220	0.96	-0.08, 1.99	87	
	Sample Size							0.0582
	More than 30	10	483	394	**1.31**	0.35, 2.27	91	
	30 and below	13	133	110	0.23	-0.35, 0.81	40	
	Study Design							0.0522
	Observational	17	569	458	**0.96**	0.21, 1.71	87	
	Interventional	6	47	46	0.04	-0.50, 0.59	0	
	Bias							0.7706
	Moderate	22	531	420	**0.77**	0.11, 1.43	84	
	Low	1	85	84	**0.90**	0.33,1.47	-	
**Neutrophils**	Phase							**0.0273**
	Acute_Subacute	3	147	112	**2.38**	0.46, 4.31	89	
	Chronic	8	194	183	0.16	-0.29, 0.60	70	
	Completeness of Injury							0.0868
	Complete	4	45	33	0.14	-0.33, 0.62	39	
	Mixed	7	296	262	**1.24**	0.08, 2.39	93	
	Sympathetic Impairment							0.0868
	Above T6	4	45	33	0.14	-0.33, 0.62	39	
	Mixed	7	296	262	**1.24**	0.08, 2.39	93	
	Presence of Females							0.2183
	All Males	6	145	135	0.26	-0.30, 0.81	76	
	With Females	5	196	160	1.34	0.30, 2.98	93	
	Age Groups							**0.0137**
	With Seniors	6	271	237	**1.59**	0.39, 2.79	89	
	Adults	5	70	58	-0.07	-0.61, 0.47	70	
	Location							0.6400
	Europe and the Americas	6	187	172	0.55	-0.04, 1.13	83	
	Asia and the Pacific	5	154	123	1.03	-0.92, 2.99	93	
	Sample Size							0.4524
	More than 30	5	280	250	1.04	-0.29, 2.37	95	
	30 and below	6	61	45	0.47	-0.22, 1.16	69	
	Study Design							0.2025
	Observational	8	318	275	**0.89**	0.10, 1.67	92	
	Interventional	3	23	20	0.16	-0.64, 0.96	0	
	Bias							0.6584
	Moderate	10	256	211	0.75	-0.03, 1.51	90	
	Low	1	85	84	**0.95**	0.48, 1.42	-	
**Lymphocytes**	Phase							**0.0197**
	Acute_Subacute	3	159	117	**-0.45**	-0.78, -0.11	73	

	Chronic	10	223	212	-0.02	-0.15, 0.10	47	
	Completeness of Injury							**0.0020**
	Complete	6	74	62	0.06	-0.06, 0.09	18	
	Mixed	7	308	267	**-0.28**	-0.47, -0.10	72	
	Sympathetic Impairment							0.1618
	Above T6	6	77	55	-0.01	-0.26, 0.24	68	
	Mixed	7	305	274	**-0.22**	-0.36, -0.07	46	
	Presence of Females							**0.0352**
	All Males	7	156	146	-0.01	-0.17, 0.15	40	
	With Females	7	226	183	**-0.28**	-0.48, -0.08	63	
	Age Groups							0.0735
	With Seniors	6	283	242	**-0.27**	-0.48, -0.06	66	
	Adults	7	99	87	-0.01	-0.20, 0.18	62	
	Location							0.9750
	Europe and North America	8	228	206	-0.15	-0.34, 0.03	81	
	Asia and the Pacific	5	154	123	-0.16	-0.30, -0.01	0	
	Sample Size							**0.0028**
	More than 30	6	301	261	**-0.28**	-0.47, -0.09	70	
	30 and below	7	81	68	0.06	-0.06, 0.17	12	
	Study Design							**0.0493**
	Observational	9	348	329	-0.18	-0.37, 0.01	75	
	Interventional	4	34	31	0.02	-0.04, 0.08	0	
	Bias							0.2750
	Moderate	12	297	245	-0.15	-0.31, 0.01	74	
	Low	1	85	84	-0.02	-0.19, 0.16	-	
**Monocytes**	Phase							**0.0146**
	Acute_Subacute	3	147	112	**0.07**	0.02, 0.12	0	
	Chronic	11	259	264	0.00	-0.03, 0.02	34	
	Completeness of Injury							0.8127
	Complete	5	63	51	0.00	-0.02, 0.03	0	
	Mixed	9	343	325	0.01	-0.04, 0.05	63	
	Sympathetics Impairment							0.6061
	Above T6	4	45	33	0.00	-0.03, 0.03	0	
	Mixed	10	361	343	0.01	-0.03, 0.05	59	
	Presence of Females							0.1812
	All Males	7	164	143	-0.01	-0.04, 0.03	38	
	With Females	7	242	233	0.03	-0.01, 0.07	43	
	Age Groups							0.1923
	With Seniors	6	271	237	0.03	-0.02, 0.08	50	
	Adults	8	135	139	-0.01	-0.04, 0.02	23	
	Location							0.9631
	Europe and North America	8	233	245	0.01	-0.03, 0.05	57	
	Asia and the Pacific	6	173	131	0.01	-0.04, 0.05	16	
	Sample Size							0.8849
	More than 30	6	308	305	0.01	-0.05, 0.06	69	
	30 and below	8	98	71	0.00	-0.02, 0.03	0	
	Study Design							0.8623
	Observational	11	383	356	0.01	-0.02, 0.04	56	
	Interventional	3	23	20	0.00	-0.09, 0.09	0	
	Bias							0.0613
	Moderate	12	293	237	0.00	-0.03, 0.03	40	
	Low	2	113	139	0.05	0.01, 0.09	0	
**IL6**[a]	Phase							0.1902
	Acute_Subacute	4	201	119	7.25	-2.61, 17.11	99	
	Chronic	9	283	244	0.65	-0.07, 1.37	87	
	Completeness of Injury							0.0708
	Complete	1	8	8	**1.16**	0.82, 1.50	-	
	Mixed	12	476	355	**2.52**	1.08, 3.96	97	
	Sympathetics Impairment							0.0758
	Above T6	4	58	48	**1.07**	0.17, 1.98	75	
	Mixed	9	426	315	**2.89**	1.1, 4.68	98	
	Presence of Females							**0.0117**

	All Males	7	247	179	0.58	-0.38, 1.53	89	
	With Females	6	237	184	**4.56**	1.61, 7.50	99	
	Age Groups							**0.004**
	With Seniors	8	361	251	**3.67**	1.72, 5.62	98	
	Adults	5	123	112	0.64	-0.05, 1.32	64	
	Location							0.451
	Europe and North America	8	260	253	3.04	-1.20,7.28	98	
	Asia and the Pacific	5	224	110	1.4	-0.89, 1.90	86	
	Sample Size							**0.01**
	More than 30	9	437	327	**3.29**	1.39, 5.19	98	
	30 and below	4	47	36	0.61	-0.11, 1.33	75	
	Bias							**0.0166**
	Moderate	13	406	280	**2.67**	1.46, 3.87	97	
	Low	1	78	83	-0.60	-1.52, 0.32	-	
	Sample Source							**0.0151**
	Serum	8	346	224	**3.39**	1.40, 5.38	98	
	Plasma	5	138	139	0.70	-0.17, 1.57	79	
**CRP**[ab]	Completeness of Injury							0.4653
	Complete	1	6	8	3.20	1.00, 5.40	-	
	Mixed	11	543	407	**2.27**	1.01, 3.53	95	
	Sympathetic Impairment							0.9636
	Above T6	2	28	19	**2.29**	0.98, 3.59	6	
	Mixed	10	521	396	**2.33**	0.98, 3.68	95	
	Presence of Females							0.8129
	All Males	9	404	332	**2.16**	1.21, 3.10	73	
	With Females	3	145	83	2.55	-0.58, 5.68	97	
	Age Groups							0.3542
	With Seniors	3	237	162	1.57	-0.40, 3.54	98	
	Adults	9	287	228	**2.77**	1.17, 4.37	85	
	Location							0.9226
	Europe and North America	9	366	326	**2.41**	1.17, 3.64	86	
	Asia and the Pacific	3	183	89	2.23	-1.12, 5.58	98	
	Sample Size							0.3336
	More than 30	10	524	374	**2.19**	0.92, 3.46	95	
	30 and below	2	25	16	**3.40**	1.30, 5.50	0	
	Bias							0.6163
	Moderate	9	313	195	**2.42**	0.77, 4.06	95	
	Low	3	236	220	**1.85**	0.45, 3.27	70	
	Sample Source							0.7910
	Serum	10	448	315	**2.90**	1.00, 4.80	96	
	Plasma	2	79	89	**2.00**	0.30, 3.60	60	

[a]All studies were observational.
[b]All studies were conducted with individuals with chronic SCI.
CRP, c-reactive protein; IL, interleukin; WMD, weighted mean difference, SCI, spinal cord injury; CI, confidence interval
All values in bold are significant values at *p-value* <0.05

Supplemental Table S14. Summary of the results of the meta-regression analysis performed for the circulating immune markers that were included in the meta-analysis of studies comparing individuals with SCI vs those without SCI.

**Circulating Immune Marker**	**Variable**	**β**	**95% CI**	***p-value***
Leukocytes	Mean Age	0.0851	0.0077, 0.1625	**0.0312**
	Mean Injury Duration	-0.1282	-0.2129, -0.0435	**0.0030**
	Percentage of Males	-0.0256	-0.0710, 0.0199	0.2703
Neutrophils	Mean Age	0.1298	0.0313, 0.2284	**0.0098**
	Mean Injury Duration	-0.1014	-0.2789, -0.0760	0.2626
	Percentage of Males	-0.0430	-0.0936, 0.0077	0.0962
Lymphocytes	Mean Age	-0.0072	-0.0239, 0.0096	0.4015
	Mean Injury Duration	0.0211	0.0045, 0.0368	**0.0130**
	Percentage of Males	0.0077	-0.0018, 0.0172	0.1104
Monocyte	Mean Age	0.0018	-0.0018, 0.0054	0.3264
	Mean Injury Duration	-0.0030	-0.0079, 0.0020	0.2443
	Percentage of Males	-0.0014	-0.0030, 0.0003	0.1083
IL6	Mean Age	-0.103	-0.2727, 0.0668	0.2345
	Mean Injury Duration	-0.2514	-0.4549, -0.0479	**0.0155**
	Percentage of Males	-0.0195	-0.1222, 0.0832	0.7097
CRP	Mean Age	-0.0870	-0.2241, 0.0502	0.2140
	Mean Injury Duration	-0.0665	-0.2227, 0.0897	0.4040
	Percentage of Males	0.0257	-0.0742, 0.1256	0.6143

β, coefficient of the slope, CI, confidence interval
All values in bold are significant values at *p-value* <0.05

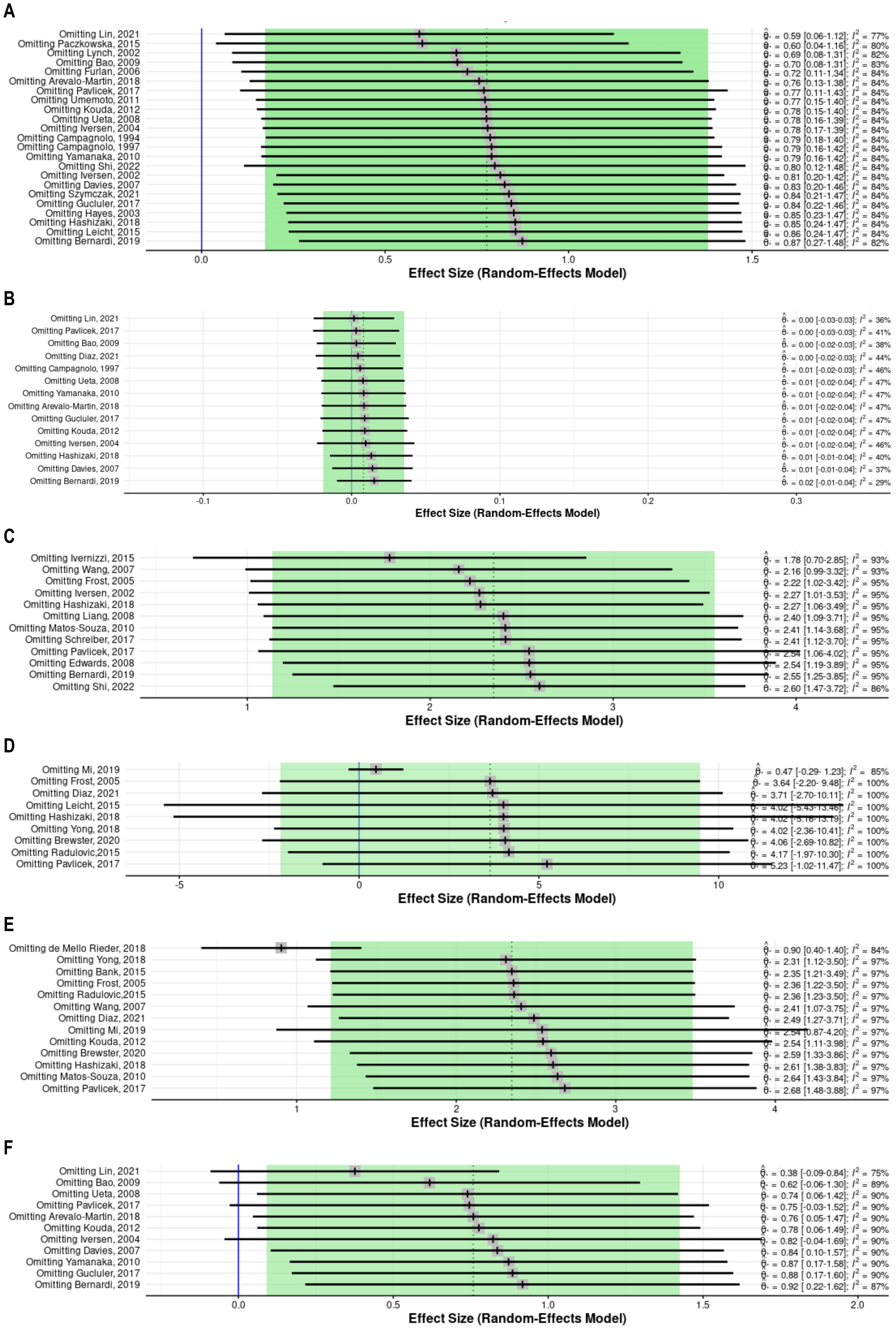
A
Omitting Lin, 2021
Omitting Paczkowska, 2015
Omitting Lynch, 2002
Omitting Bao, 2009
Omitting Furlan, 2006
Omitting Arevalo-Martin, 2018
Omitting Pavlicek, 2017
Omitting Umemoto, 2011
Omitting Kouda, 2012
Omitting Ueta, 2008
Omitting Iversen, 2004
Omitting Campagnolo, 1994
Omitting Campagnolo, 1997
Omitting Yamanaka, 2010
Omitting Shi, 2022
Omitting Iversen, 2002
Omitting Davies, 2007
Omitting Szymczak, 2021
Omitting Gucluler, 2017
Omitting Hayes, 2003
Omitting Hashizaki, 2018
Omitting Leicht, 2015
Omitting Bernardi, 2019
θ̂ = 0.59 [0.06-1.12]; I² = 77%
θ̂ = 0.60 [0.04-1.16]; I² = 80%
θ̂ = 0.69 [0.08-1.31]; I² = 82%
θ̂ = 0.70 [0.08-1.31]; I² = 83%
θ̂ = 0.72 [0.11-1.34]; I² = 84%
θ̂ = 0.76 [0.13-1.38]; I² = 84%
θ̂ = 0.77 [0.11-1.43]; I² = 84%
θ̂ = 0.77 [0.15-1.40]; I² = 84%
θ̂ = 0.78 [0.15-1.40]; I² = 84%
θ̂ = 0.78 [0.16-1.39]; I² = 84%
θ̂ = 0.78 [0.17-1.39]; I² = 84%
θ̂ = 0.79 [0.18-1.40]; I² = 84%
θ̂ = 0.79 [0.16-1.42]; I² = 84%
θ̂ = 0.79 [0.16-1.42]; I² = 84%
θ̂ = 0.80 [0.12-1.48]; I² = 84%
θ̂ = 0.81 [0.20-1.42]; I² = 84%
θ̂ = 0.83 [0.20-1.46]; I² = 84%
θ̂ = 0.84 [0.21-1.47]; I² = 84%
θ̂ = 0.84 [0.22-1.46]; I² = 84%
θ̂ = 0.85 [0.23-1.47]; I² = 84%
θ̂ = 0.85 [0.24-1.47]; I² = 84%
θ̂ = 0.86 [0.24-1.47]; I² = 84%
θ̂ = 0.87 [0.27-1.48]; I² = 82%
0.0
0.5
1.0
1.5
Effect Size (Random-Effects Model)
B
Omitting Lin, 2021
Omitting Pavlicek, 2017
Omitting Bao, 2009
Omitting Diaz, 2021
Omitting Campagnolo, 1997
Omitting Ueta, 2008
Omitting Yamanaka, 2010
Omitting Arevalo-Martin, 2018
Omitting Gucluler, 2017
Omitting Kouda, 2012
Omitting Iversen, 2004
Omitting Hashizaki, 2018
Omitting Davies, 2007
Omitting Bernardi, 2019
θ̂ = 0.00 [-0.03-0.03]; I² = 36%
θ̂ = 0.00 [-0.03-0.03]; I² = 41%
θ̂ = 0.00 [-0.02-0.03]; I² = 38%
θ̂ = 0.00 [-0.02-0.03]; I² = 44%
θ̂ = 0.01 [-0.02-0.03]; I² = 46%
θ̂ = 0.01 [-0.02-0.04]; I² = 47%
θ̂ = 0.01 [-0.02-0.04]; I² = 47%
θ̂ = 0.01 [-0.02-0.04]; I² = 47%
θ̂ = 0.01 [-0.02-0.04]; I² = 47%
θ̂ = 0.01 [-0.02-0.04]; I² = 47%
θ̂ = 0.01 [-0.02-0.04]; I² = 46%
θ̂ = 0.01 [-0.01-0.04]; I² = 40%
θ̂ = 0.01 [-0.01-0.04]; I² = 37%
θ̂ = 0.02 [-0.01-0.04]; I² = 29%
-0.1
0.0
0.1
0.2
0.3
Effect Size (Random-Effects Model)
C
Omitting Ivernizzi, 2015
Omitting Wang, 2007
Omitting Frost, 2005
Omitting Iversen, 2002
Omitting Hashizaki, 2018
Omitting Liang, 2008
Omitting Matos-Souza, 2010
Omitting Schreiber, 2017
Omitting Pavlicek, 2017
Omitting Edwards, 2008
Omitting Bernardi, 2019
Omitting Shi, 2022
θ̂ = 1.78 [0.70-2.85]; I² = 93%
θ̂ = 2.16 [0.99-3.32]; I² = 93%
θ̂ = 2.22 [1.02-3.42]; I² = 95%
θ̂ = 2.27 [1.01-3.53]; I² = 95%
θ̂ = 2.27 [1.06-3.49]; I² = 95%
θ̂ = 2.40 [1.09-3.71]; I² = 95%
θ̂ = 2.41 [1.14-3.68]; I² = 95%
θ̂ = 2.41 [1.12-3.70]; I² = 95%
θ̂ = 2.54 [1.06-4.02]; I² = 95%
θ̂ = 2.54 [1.19-3.89]; I² = 95%
θ̂ = 2.55 [1.25-3.85]; I² = 95%
θ̂ = 2.60 [1.47-3.72]; I² = 86%
1
2
3
4
Effect Size (Random-Effects Model)
D
Omitting Mi, 2019
Omitting Frost, 2005
Omitting Diaz, 2021
Omitting Leicht, 2015
Omitting Hashizaki, 2018
Omitting Yong, 2018
Omitting Brewster, 2020
Omitting Radulovic,2015
Omitting Pavlicek, 2017
θ̂ = 0.47 [-0.29- 1.23]; I² = 85%
θ̂ = 3.64 [-2.20- 9.48]; I² = 100%
θ̂ = 3.71 [-2.70-10.11]; I² = 100%
θ̂ = 4.02 [-5.43-13.46]; I² = 100%
θ̂ = 4.02 [-5.16-13.19]; I² = 100%
θ̂ = 4.02 [-2.36-10.41]; I² = 100%
θ̂ = 4.06 [-2.69-10.82]; I² = 100%
θ̂ = 4.17 [-1.97-10.30]; I² = 100%
θ̂ = 5.23 [-1.02-11.47]; I² = 100%
-5
0
5
10
Effect Size (Random-Effects Model)
E
Omitting de Mello Rieder, 2018
Omitting Yong, 2018
Omitting Bank, 2015
Omitting Frost, 2005
Omitting Radulovic,2015
Omitting Wang, 2007
Omitting Diaz, 2021
Omitting Mi, 2019
Omitting Kouda, 2012
Omitting Brewster, 2020
Omitting Hashizaki, 2018
Omitting Matos-Souza, 2010
Omitting Pavlicek, 2017
θ̂ = 0.90 [0.40-1.40]; I² = 84%
θ̂ = 2.31 [1.12-3.50]; I² = 97%
θ̂ = 2.35 [1.21-3.49]; I² = 97%
θ̂ = 2.36 [1.22-3.50]; I² = 97%
θ̂ = 2.36 [1.23-3.50]; I² = 97%
θ̂ = 2.41 [1.07-3.75]; I² = 97%
θ̂ = 2.49 [1.27-3.71]; I² = 97%
θ̂ = 2.54 [0.87-4.20]; I² = 97%
θ̂ = 2.54 [1.11-3.98]; I² = 97%
θ̂ = 2.59 [1.33-3.86]; I² = 97%
θ̂ = 2.61 [1.38-3.83]; I² = 97%
θ̂ = 2.64 [1.43-3.84]; I² = 97%
θ̂ = 2.68 [1.48-3.88]; I² = 97%
1
2
3
4
Effect Size (Random-Effects Model)
F
Omitting Lin, 2021
Omitting Bao, 2009
Omitting Ueta, 2008
Omitting Pavlicek, 2017
Omitting Arevalo-Martin, 2018
Omitting Kouda, 2012
Omitting Iversen, 2004
Omitting Davies, 2007
Omitting Yamanaka, 2010
Omitting Gucluler, 2017
Omitting Bernardi, 2019
θ̂ = 0.38 [-0.09-0.84]; I² = 75%
θ̂ = 0.62 [-0.06-1.30]; I² = 89%
θ̂ = 0.74 [0.06-1.42]; I² = 90%
θ̂ = 0.75 [-0.03-1.52]; I² = 90%
θ̂ = 0.76 [0.05-1.47]; I² = 90%
θ̂ = 0.78 [0.06-1.49]; I² = 90%
θ̂ = 0.82 [-0.04-1.69]; I² = 90%
θ̂ = 0.84 [0.10-1.57]; I² = 90%
θ̂ = 0.87 [0.17-1.58]; I² = 90%
θ̂ = 0.88 [0.17-1.60]; I² = 90%
θ̂ = 0.92 [0.22-1.62]; I² = 87%
0.0
0.5
1.0
1.5
2.0
Effect Size (Random-Effects Model)

G

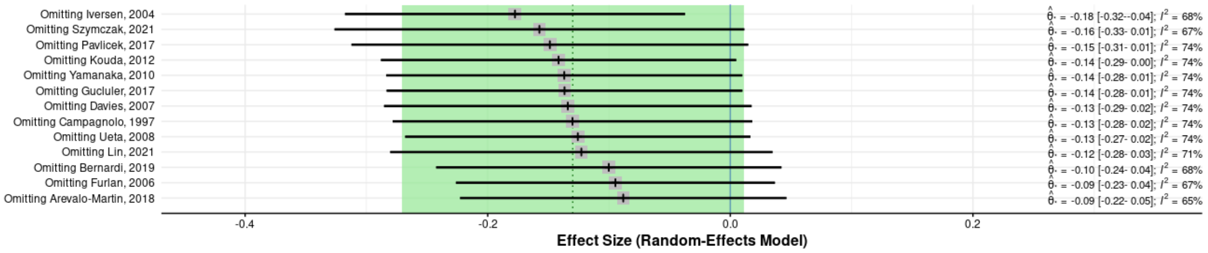

Supplemental Figure S1. The results of the leave one out analysis for the circulating leukocytes [29,33, 77, 28, 14, 35, 48, 4, 5, 1, 49, 37, 38, 6, 34, 87, 40, 2, 43, 45, 3, 10, 90] (A), monocytes [29, 48, 28, 41, 38, 1, 6, 35, 43, 5, 49, 3, 40, 90] (B), CRP [88, 57, 66, 87, 3, 68, 61, 89, 48, 86, 90, 34] (C), TNFα [62, 66, 41, 10, 3, 54, 70, 67, 48] (D), IL6 [55, 54, 52, 66, 67, 57, 41, 62, 5, 70, 3, 61, 48] (E), neutrophils [29, 28, 1, 48, 35, 5, 39, 40, 6, 43, 90] (F), and lymphocytes [49, 2, 48, 5, 6, 43, 40, 38, 1, 29, 90, 14, 35] (G) among the studies comparing individuals with SCI vs those without SCI.

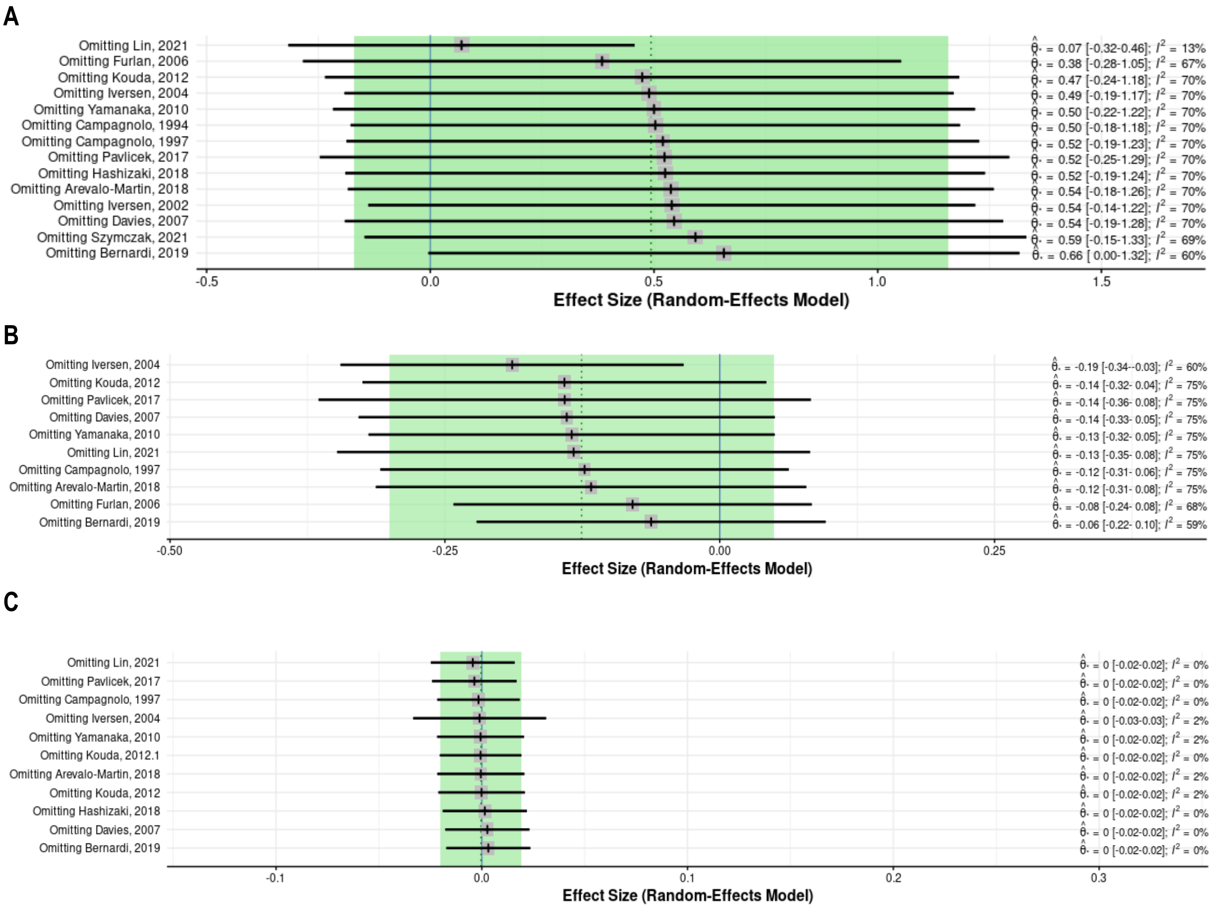

Supplemental Figure S2. The results of the leave one out analysis for the circulating leukocytes [29, 14, 5, 49, 6, 37, 38, 48, 3, 35, 87, 40, 2, 90] (A), lymphocytes [49, 5, 48, 40, 6, 29, 38, 35, 14, 90] (B), and monocytes [29, 48, 38, 49, 6, 5, 35, 3, 40, 90] (C) among the studies comparing individuals with tetraplegia vs those without SCI.

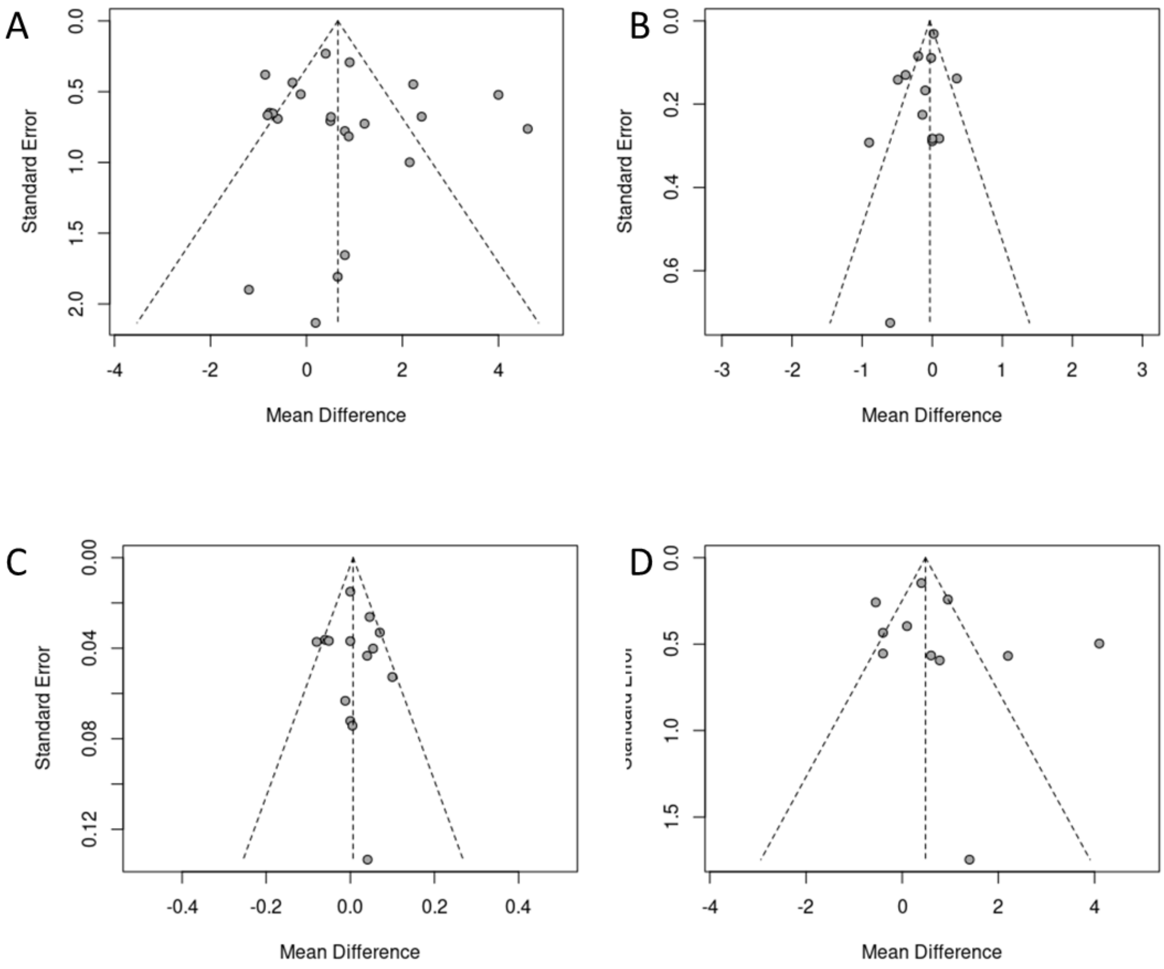

Supplemental Figure S3. The funnel plot analysis for the circulating leukocytes (A), neutrophils (B), lymphocytes (C), and monocytes (D) among the studies comparing individuals with SCI vs those without SCI.

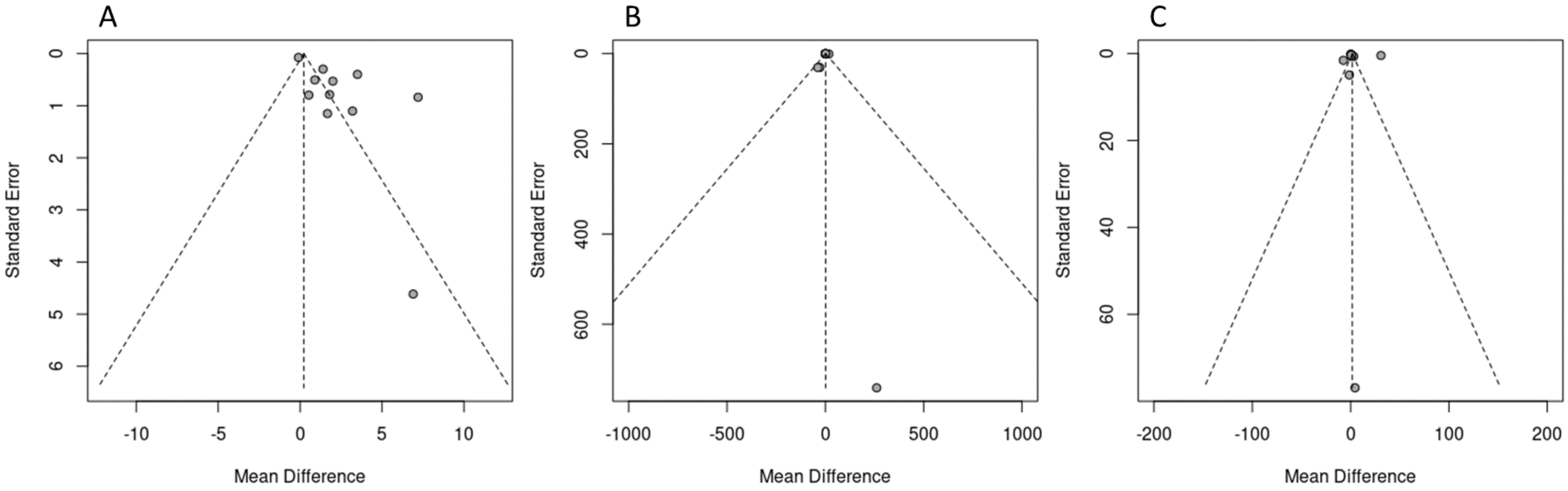

Supplemental Figure S4. The funnel plot analysis for the circulating CRP (A), IL6 (B), and TNFα (C) among the studies comparing individuals with SCI vs those without SCI.

Supplemental Table S15. Summary of the Egger test results of circulating immune markers among individuals with SCI vs those without SCI

Immune Marker	Intercept	95% CI	*p-value*
Leukocytes	0.512	-1.62, 2.64	0.6427
Monocytes	0.190	-1.34, 1.72	0.8117
Lymphocytes	-1.114	-2.50, 0.27	0.1438
Neutrophil	1.423	-2.04, 4.89	0.4417
CRP	3.867	1.83, 3.73	0.0039
IL6	0.884	-3.69, 5.46	0.7120
TNFα	8.508	-14.69, 31.71	0.4956

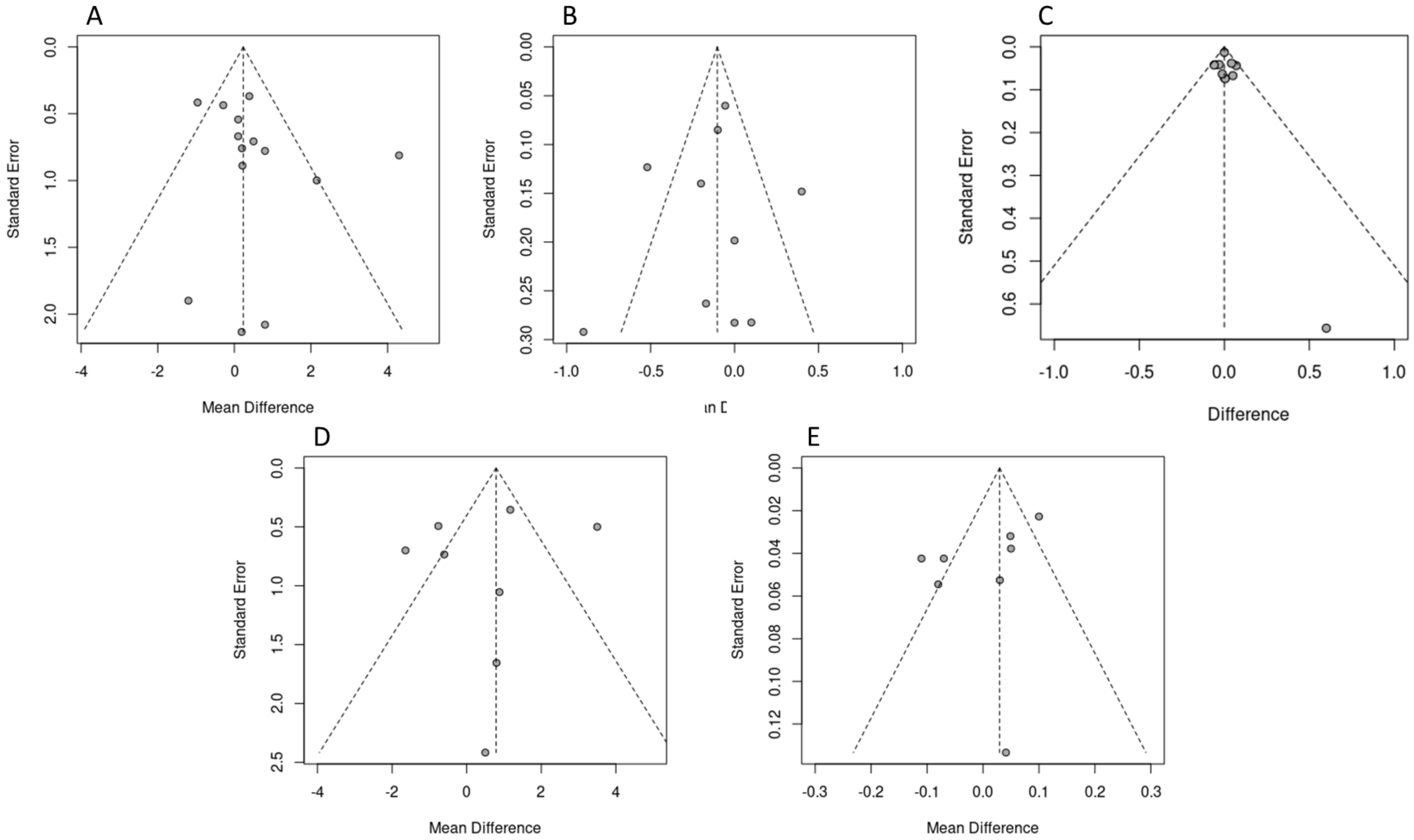

Supplemental Figure S5. The funnel plot analysis for the circulating leukocytes (A), lymphocytes (B), and monocytes (C) among the studies comparing individuals with tetraplegia vs those without SCI and the funnel plot analysis for the circulating leukocytes (D) and monocytes (E) among the studies comparing individuals with paraplegia vs those without SCI.

Supplemental Table S16. Summary of the Egger test results of circulating immune markers among individuals with tetraplegia vs those without SCI

	Intercept	95% CI	*p-value*
Tetraplegia			
Leukocytes	1.27	-0.80, 3.34	0.25
Monocytes	0.24	-0.71,1.19	0.65
Lymphocytes	-0.49	-2.94, 1.96	0.70
Paraplegia			
Leukocytes	-1.20	-5.68, 3.29	0.62
Monocytes	-2.90	-6.31,0.52	0.14

References

1. Ueta M, Furusawa K, Takahashi M, Akatsu Y, Nakamura T, Tajima F. Attenuation of natural killer cell activity during 2-h exercise in individuals with spinal cord injuries. Spinal cord. 2008;46(1):26-32.
2. Szymczak L, Podgorski T, Domaszewska K. Comparison of the levels of hematological parameters at rest and after maximum exercise between physically active people with spinal cord injury and able-bodied people. International Journal of Environmental Research and Public Health. 2021;18(23) (no pagination).
3. Hashizaki T, Nishimura Y, Teramura K, Umemoto Y, Shibasaki M, Leicht CA, Kouda K, Tajima F. Differences in serum IL-6 response after 1 degreeC rise in core body temperature in individuals with spinal cord injury and cervical spinal cord injury during local heat stress. International Journal of Hyperthermia. 2018;35(1):541-7.
4. Umemoto Y, Furusawa K, Kouda K, Sasaki Y, Kanno N, Kojima D, Tajima F. Plasma IL-6 levels during arm exercise in persons with spinal cord injury. Spinal Cord. 2011;49(12):1182-7.
5. Kouda K, Furusawa K, Sugiyama H, Sumiya T, Ito T, Tajima F, Shimizu K. Does 20-min arm crank ergometer exercise increase plasma interleukin-6 in individuals with cervical spinal cord injury? Eur J Appl Physiol. 2012;112(2):597-604.
6. Yamanaka M, Furusawa K, Sugiyama H, Goto M, Kinoshita T, Kanno N, Takaoka K, Tajima F. Impaired immune response to voluntary arm-crank ergometer exercise in patients with cervical spinal cord injury. Spinal Cord. 2010;48(10):734-9.
7. Chen SF, Chang CH, Kuo HC. Effect of detrusor botulinum toxin a injection on urothelial dysfunction in patients with chronic spinal cord injury: a clinical and immunohistochemistry study before and after treatment. Spinal Cord. 2016;54(10):889-94.
8. Chen SF, Chang CH, Kuo HC. Clinical Efficacy and Changes of Urothelial Dysfunction after Repeated Detrusor Botulinum Toxin A Injections in Chronic Spinal Cord-Injured Bladder. Toxins. 2016;8(6).
9. Jiang YH, Liu HT, Kuo HC. Urothelial dysfunction and chronic inflammation in patients with spinal cord injuries at different levels and correlation with urodynamic findings. Neurourology & Urodynamics. 2015;34(8):757-62.
10. Leicht CA, Kouda K, Umemoto Y, Banno M, Kinoshita T, Moriki T, Nakamura T, Bishop NC, Goosey-Tolfrey VL, Tajima F. Hot water immersion induces an acute cytokine response in cervical spinal cord injury. Eur J Appl Physiol. 2015;115(11):2243-52.
11. Leicht CA, Bishop NC, Goosey-Tolfrey VL. Mucosal immune responses to treadmill exercise in elite wheelchair athletes. Medicine and Science in Sports and Exercise. 2011;43(8):1414-21.
12. Kliesch WF, Cruse JM, Lewis RE, Bishop GR, Brackin B, Lampton JA. Restoration of depressed immune function in spinal cord injury patients receiving rehabilitation therapy. Paraplegia. 1996;34(2):82-90.
13. Wichmann TO, Kasch H, Dyrskog S, Hoy K, Moller BK, Krog J, Hviid CVB, Hoffmann HJ, Rasmussen MM. The inflammatory response and blood-spinal cord barrier integrity in traumatic spinal cord injury: a prospective pilot study. Acta Neurochirurgica. 2022;164(12):3143-53.
14. Furlan JC, Krassioukov AV, Fehlings MG. Hematologic abnormalities within the first week after acute isolated traumatic cervical spinal cord injury: A case-control cohort study. Spine. 2006;31(23):2674-83.
15. Heller RA, Raven TF, Swing T, Kunzmann K, Daniel V, Haubruck P, Akbar M, Grützner PA, Schmidmaier G, Biglari B, et al. CCL-2 as a possible early marker for remission after traumatic spinal cord injury. Spinal Cord. 2017;55(11):1002-9.

16. Aird IA, Vince GS, Bates MD, Johnson PM, Lewis-Jones ID. Leukocytes in semen from men with spinal cord injuries. Fertility & Sterility. 1999;72(1):97-103.
17. Basu S, Lynne CM, Ruiz P, Aballa TC, Ferrell SM, Brackett NL. Cytofluorographic identification of activated T-cell subpopulations in the semen of men with spinal cord injuries. Journal of Andrology. 2002;23(4):551-6.
18. Brackett NL, Ibrahim E, Grotas JA, Aballa TC, Lynne CM. Higher sperm DNA damage in semen from men with spinal cord injuries compared with controls. Journal of Andrology. 2008;29(1):93-9; discussion 100-1.
19. Brackett NL, Cohen DR, Ibrahim E, Aballa TC, Lynne CM. Neutralization of cytokine activity at the receptor level improves sperm motility in men with spinal cord injuries. Journal of Andrology. 2007;28(5):717-21.
20. da Silva BF, Borrelli M, Jr., Fariello RM, Restelli AE, Del Giudice PT, Spaine DM, Bertolla RP, Cedenho AP. Is sperm cryopreservation an option for fertility preservation in patients with spinal cord injury-induced anejaculation? Fertility & Sterility. 2010;94(2):564-73.
21. da Silva BF, Souza GH, lo Turco EG, Del Giudice PT, Soler TB, Spaine DM, Borrelli Junior M, Gozzo FC, Pilau EJ, Garcia JS, et al. Differential seminal plasma proteome according to semen retrieval in men with spinal cord injury. Fertil Steril. 2013;100(4):959-69.
22. Padron OF, Brackett NL, Sharma RK, Lynne CM, Thomas AJ, Jr., Agarwal A. Seminal reactive oxygen species and sperm motility and morphology in men with spinal cord injury. Fertil Steril. 1997;67(6):1115-20.
23. Salsabili N, Ziaei AM, Taheri M, Akbari F, Jalaie S. Impact of sperm collection methods on sperm parameters in spinal cord injured men and compared to normal controls in ICSI program. Sexuality and Disability. 2006;24(3):141-9.
24. Trabulsi EJ, Shupp-Byrne D, Sedor J, Hirsch IH. Leukocyte subtypes in electroejaculates of spinal cord injured men. Archives of Physical Medicine & Rehabilitation. 2002;83(1):31-4.
25. Basu S, Aballa TC, Ferrell SM, Lynne CM, Brackett NL. Inflammatory cytokine concentrations are elevated in seminal plasma of men with spinal cord injuries. Journal of Andrology. 2004;25(2):250-4.
26. Zhang X, Ibrahim E, de Rivero Vaccari JP, Lotocki G, Aballa TC, Dietrich WD, Keane RW, Lynne CM, Brackett NL. Involvement of the inflammasome in abnormal semen quality of men with spinal cord injury. Fertility & Sterility. 2013;99(1):118-24.e2.
27. Bao F, Bailey CS, Gurr KR, Bailey SI, Rosas-Arellano MP, Brown A, Dekaban GA, Weaver LC. Human spinal cord injury causes specific increases in surface expression of beta integrins on leukocytes. Journal of Neurotrauma. 2011;28(2):269-80.
28. Bao F, Bailey CS, Gurr KR, Bailey SI, Rosas-Arellano MP, Dekaban GA, Weaver LC. Increased oxidative activity in human blood neutrophils and monocytes after spinal cord injury. Experimental Neurology. 2009;215(2):308-16.
29. Lin S, Li D, Zhou Z, Xu C, Mei X, Tian H. Therapy of spinal cord injury by zinc modified gold nanoclusters via immune-suppressing strategies. Journal of Nanobiotechnology. 2021;19(1) (no pagination).
30. Riegger T, Conrad S, Schluesener HJ, Kaps HP, Badke A, Baron C, Gerstein J, Dietz K, Abdizahdeh M, Schwab JM. Immune depression syndrome following human spinal cord injury (SCI): a pilot study. Neuroscience. 2009;158(3):1194-9.
31. Heller RA, Seelig J, Crowell HL, Pilz M, Haubruck P, Sun Q, Schomburg L, Daniel V, Moghaddam A, Biglari B. Predicting neurological recovery after traumatic spinal cord injury by time-resolved analysis of monocyte subsets. Brain. 2021;144(10):3159-74.
32. Xu L, Zhang Y, Zhang R, Zhang H, Song P, Ma T, Li Y, Wang X, Hou X, Li Q, et al. Elevated plasma BDNF levels are correlated with NK cell activation in patients with traumatic spinal cord injury. International Immunopharmacology. 2019;74:105722.
33. Paczkowska E, Rogińska D, Pius-Sadowska E, Jurewicz A, Piecyk K, Safranow K, Dziedziejko V, Grzegrzółka R, Bohatyrewicz A, Machaliński B. Evidence for proangiogenic cellular and humoral systemic response in patients with acute onset of spinal cord injury. J Spinal Cord Med. 2015;38(6):729-44.
34. Shi H, Xie L, Xu W, Cao S, Chen Y. Nogo-A Is a Potential Prognostic Marker for Spinal Cord Injury. Dis Markers. 2022;2022:2141854.
35. Arevalo-Martin A, Grassner L, Garcia-Ovejero D, Paniagua-Torija B, Barroso-Garcia G, Arandilla AG, Mach O, Turrero A, Vargas E, Alcobendas M, et al. Elevated Autoantibodies in Subacute Human Spinal Cord Injury Are Naturally Occurring Antibodies. Frontiers in Immunology. 2018;9:2365.
36. Grassner L, Klein B, Garcia-Ovejero D, Mach O, Scheiblhofer S, Weiss R, Vargas-Baquero E, Kramer JLK, Leister I, Rohde E, et al. Systemic Immune Profile Predicts the Development of Infections in Patients with Spinal Cord Injuries. J Neurotrauma. 2022;39(23-24):1678-86.

37. Campagnolo DI, Keller SE, DeLisa JA, Glick TJ, Sipski ML, Schleifer SJ. Alteration of immune system function in tetraplegics. A pilot study. American Journal of Physical Medicine & Rehabilitation. 1994;73(6):387-93.
38. Campagnolo DI, Bartlett JA, Keller SE, Sanchez W, Oza R. Impaired phagocytosis of Staphylococcus aureus in complete tetraplegics. American Journal of Physical Medicine & Rehabilitation. 1997;76(4):276-80.
39. Campagnolo DI, Dixon D, Schwartz J, Bartlett JA, Keller SE. Altered innate immunity following spinal cord injury. Spinal Cord. 2008;46(7):477-81.
40. Davies AL, Hayes KC, Dekaban GA. Clinical correlates of elevated serum concentrations of cytokines and autoantibodies in patients with spinal cord injury. Archives of Physical Medicine & Rehabilitation. 2007;88(11):1384-93.
41. Diaz D, Lopez-Dolado E, Haro S, Monserrat J, Martinez-Alonso C, Balomeros D, Albillos A, Alvarez-Mon M. Systemic Inflammation and the Breakdown of Intestinal Homeostasis Are Key Events in Chronic Spinal Cord Injury Patients. International Journal of Molecular Sciences. 2021;22(2):13.
42. Formisano R, Grelli S, Matteucci C, Santilli V, Vinicola V, Scivoletto G, Castellano V, D'Agostini C, Mastino A, Favalli C. Immunological and endocrinological disturbances in patients after prolonged coma following head injury. European Journal of Neurology. 1998;5(2):151-8.
43. Gucluler G, Adiguzel E, Gungor B, Kahraman T, Gursel M, Yilmaz B, Gursel I. Impaired toll like receptor-7 and 9 induced immune activation in chronic spinal cord injured patients contributes to immune dysfunction. PLoS ONE. 2017;12(2) (no pagination).
44. Kanyilmaz S, Hepguler S, Atamaz FC, Gokmen NM, Ardeniz O, Sin A. Phagocytic and oxidative burst activity of neutrophils in patients with spinal cord injury. Archives of Physical Medicine & Rehabilitation. 2013;94(2):369-74.
45. Hayes KC, Hull TC, Delaney GA, Potter PJ, Sequeira KA, Campbell K, Popovich PG. Elevated serum titers of proinflammatory cytokines and CNS autoantibodies in patients with chronic spinal cord injury. Journal of Neurotrauma. 2002;19(6):753-61.
46. Pan SC, Hsieh SM, Wang YH, Chiang BL, Huang TS, Chang SC. In vitro maturation potential of monocyte-derived dendritic cells is impaired in patients with spinal cord injury: a case-control study. Archives of Physical Medicine & Rehabilitation. 2005;86(5):974-8.
47. Monahan R, Stein A, Gibbs K, Bank M, Bloom O. Circulating T cell subsets are altered in individuals with chronic spinal cord injury. Immunologic Research. 2015;63(1-3):3-10.
48. Pavlicek D, Krebs J, Capossela S, Bertolo A, Engelhardt B, Pannek J, Stoyanov J. Immunosenescence in persons with spinal cord injury in relation to urinary tract infections -a cross-sectional study. Immunity and Ageing. 2017;14(1) (no pagination).
49. Iversen PO, Nicolaysen A, Hjeltnes N, Nja A, Benestad HB. Preserved granulocyte formation and function, as well as bone marrow innervation, in subjects with complete spinal cord injury. British Journal of Haematology. 2004;126(6):870-7.
50. Hsieh SM, Wang YH, Chang SC, Huang TS. Low dose HIV-1 Tat improves the defective nuclear factor (NF)-kappaB activity of dendritic cells from persons with spinal cord injury. Cellular Immunology. 2009;257(1-2):105-10.
51. Pang E, Ghosh S, Chemmanam T, Grove C, Phillips T. Cerebral arterial and venous thrombosis due to COVID-19 vaccine-induced immune thrombotic thrombocytopenia. BMJ Case Reports. 2022;15(1):18.
52. Bank M, Stein A, Sison C, Glazer A, Jassal N, McCarthy D, Shatzer M, Hahn B, Chugh R, Davies P, et al. Elevated circulating levels of the pro-inflammatory cytokine macrophage migration inhibitory factor in individuals with acute spinal cord injury. Archives of Physical Medicine & Rehabilitation. 2015;96(4):633-44.
53. Huang W, Vodovotz Y, Kusturiss MB, Barclay D, Greenwald K, Boninger ML, Coen PM, Brienza D, Sowa G. Identification of distinct monocyte phenotypes and correlation with circulating cytokine profiles in acute response to spinal cord injury: a pilot study. Pm r. 2014;6(4):332-41.
54. Yong Z, Cai-Liang S, Fu-Long D, Ren-Jie H, Peng G. Correlation of cytokine levels in the peripheral blood within 24 hours after cervical spinal cord injury with the american spinal injury association impairment scale: A comparative study. [Chinese]. Chinese Journal of Tissue Engineering Research. 2018;22(24):3824-30.
55. de Mello Rieder M, Oses JP, Kutchak FM, Sartor M, Cecchini A, Rodolphi MS, Wiener CD, Kopczynski A, Muller AP, Strogulski NR, et al. Serum Biomarkers and Clinical Outcomes in Traumatic Spinal Cord Injury: Prospective Cohort Study. World Neurosurgery. 2019;122:e1028-e36.
56. Liu SQ, Ma YG, Peng H, Fan L. Monocyte chemoattractant protein-1 level in serum of patients with acute spinal cord injury. Chinese Journal of Traumatology - English Edition. 2005;8(4):216-9.

57. Wang TD, Wang YH, Huang TS, Su TC, Pan SL, Chen SY. Circulating levels of markers of inflammation and endothelial activation are increased in men with chronic spinal cord injury. Journal of the Formosan Medical Association. 2007;106(11):919-28.
58. Stein A, Panjwani A, Sison C, Rosen L, Chugh R, Metz C, Bank M, Bloom O. Pilot study: elevated circulating levels of the proinflammatory cytokine macrophage migration inhibitory factor in patients with chronic spinal cord injury. Archives of Physical Medicine & Rehabilitation. 2013;94(8):1498-507.
59. Chen Y, Liang L, Cao S, Hou G, Zhang Q, Ma H, Shi B. Serum CCL21 as a Potential Biomarker for Cognitive Impairment in Spinal Cord Injury. Biomed Res Int. 2020;2020:6692802.
60. Chen Y, Wang D, Cao S, Hou G, Ma H, Shi B. Association between Serum IL-37 and Spinal Cord Injury: A Prospective Observational Study. BioMed Research International. 2020;2020:6664313.
61. Matos-Souza JR, Pithon KR, Ozahata TM, Oliveira RT, Teo FH, Blotta MH, Cliquet A, Jr., Nadruz W, Jr. Subclinical atherosclerosis is related to injury level but not to inflammatory parameters in spinal cord injury subjects. Spinal Cord. 2010;48(10):740-4.
62. Mi S, Wu Y, Hong Z, Wang Z, Feng X, Zheng G. Expression of TLR4/MyD88/NF-kappaB pathway genes and its related inflammatory factors in secondary spinal cord injury. [Chinese]. Zhejiang da xue xue bao. 2019;Yi xue ban = Journal of Zhejiang University. Medical sciences. 48(6):609-16.
63. Segal JL, Brunnemann SR. Circulating levels of soluble interleukin 2 receptors are elevated in the sera of humans with spinal cord injury. The Journal of the American Paraplegia Society. 1993;16(1):30-3.
64. Baria MR, Miller MM, Burner T, Hake T, Kim D, Magnussen R, Durgam S. Platelet-Rich Plasma Content of Active Spinal Cord Injured Patients: A Controlled Laboratory Study. American Journal of Physical Medicine & Rehabilitation. 2021;100(7):651-5.
65. Rogeri PS, Costa Rosa LFBP. Plasma glutamine concentration in spinal cord injured patients. Life Sciences. 2005;77(19):2351-60.
66. Frost F, Roach MJ, Kushner I, Schreiber P. Inflammatory C-reactive protein and cytokine levels in asymptomatic people with chronic spinal cord injury. Archives of Physical Medicine & Rehabilitation. 2005;86(2):312-7.
67. Radulovic M, Bauman WA, Wecht JM, LaFountaine M, Kahn N, Hobson J, Singh K, Renzi C, Yen C, Schilero GJ. Biomarkers of inflammation in persons with chronic tetraplegia. J Breath Res. 2015;9(3):036001.
68. Liang H, Mojtahedi MC, Chen D, Braunschweig CL. Elevated C-reactive protein associated with decreased high-density lipoprotein cholesterol in men with spinal cord injury. Archives of Physical Medicine & Rehabilitation. 2008;89(1):36-41.
69. Wang YH, Chen SY, Wang TD, Hwang BS, Huang TS, Su TC. The relationships among serum glucose, albumin concentrations and carotid atherosclerosis in men with spinal cord injury. Atherosclerosis. 2009;206(2):528-34.
70. Brewster LM, Coombs GB, Garcia VP, Hijmans JG, DeSouza NM, Stockelman KA, Barak OF, Mijacika T, Dujic Z, Greiner JJ, et al. Effects of circulating extracellular microvesicles from spinal cord-injured adults on endothelial cell function. Clinical Science. 2020;34(7):777.
71. Palmers I, Ydens E, Put E, Depreitere B, Bongers-Janssen H, Pickkers P, Hendrix S, Somers V. Antibody profiling identifies novel antigenic targets in spinal cord injury patients. Journal of Neuroinflammation. 2016;13(1):243.
72. Shnawa A, Lee S, Papatheodorou A, Gibbs K, Stein A, Morrison D, Bloom O. Elevated levels of IgA and IgG2 in individuals with chronic spinal cord injury. Journal of Spinal Cord Medicine. 2022;45(5):728-38.
73. Chinigo AS, Rosman AS, Spungen AM, Drexler HJ, Bauman WA. Prevalence of viral hepatitis B in an urban veteran spinal cord injured population. Journal of Spinal Cord Medicine. 1996;19(2):71-7.
74. Everall PH, Morris CA, Morris DF. Antibodies to Candida albicans in hospital patients with and without spinal injury and in normal men and women. Journal of Clinical Pathology. 1974;27(9):722-8.
75. Trautner BW, Atmar RL, Hulstrom A, Darouiche RO. Inactivated influenza vaccination for people with spinal cord injury. Archives of Physical Medicine & Rehabilitation. 2004;85(11):1886-9.
76. Rosman AS, Chinigo AS, Spungen AM, Drexler HJ, Bauman WA. Viral hepatitis in patients with spinal cord injury is explained by known risk factors. The journal of spinal cord medicine. 1998;21(1):25-31.
77. Lynch AC, Palmer C, Lynch AC, Anthony A, Roake JA, Frye J, Frizelle FA. Nutritional and immune status following spinal cord injury: a case controlled study. Spinal Cord. 2002;40(12):627-30.

78. Vaidyanathan S, McDicken IW, Soni BM, Singh G, Sett P, Husin NM. Secretory immunoglobulin A in the vesical urothelium of patients with neuropathic bladder - An immunohistochemical study. Spinal Cord. 2000;38(6):378-81.
79. Gallego Gómez J, Jiménez Cruz JF, Mompo Sanchís JA, Fernández Zuazu J, Martínez Agulló E, Beneyto Juan M. [Urinary immunoglobulins in spinal cord lesions]. Arch Esp Urol. 1983;36(4):269-79.
80. Fraussen J, Beckers L, van Laake-Geelen CCM, Depreitere B, Deckers J, Cornips EMJ, Peuskens D, Somers V. Altered Circulating Immune Cell Distribution in Traumatic Spinal Cord Injury Patients in Relation to Clinical Parameters. Frontiers in Immunology. 2022;13 (no pagination).
81. Chernykh ER, Shevela EY, Leplina OY, Tikhonova MA, Ostanin AA, Kulagin AD, Pronkina NV, Muradov Zh M, Stupak VV, Kozlov VA. Characteristics of bone marrow cells under conditions of impaired innervation in patients with spinal trauma. Bulletin of Experimental Biology and Medicine. 2006;141(1):117-20.
82. Iversen PO, Hjeltnes N, Holm B, Flatebo T, Strom-Gundersen I, Ronning W, Stanghelle J, Benestad HB. Depressed immunity and impaired proliferation of hematopoietic progenitor cells in patients with complete spinal cord injury. Blood. 2000;96(6):2081-3.
83. Hassanshahi G, Amin M, Shunmugavel A, Vazirinejad R, Vakilian A, Sanji M, Shamsizadeh A, RafatPanah H, Poor NM, Moosavi SR, et al. Temporal expression profile of CXC chemokines in serum of patients with spinal cord injury. Neurochemistry International. 2013;63(5):363-7.
84. Lieberman J, Goff D, Jr., Hammond F, Schreiner P, James Norton H, Dulin M, Zhou X, Steffen L. Dietary intake relative to cardiovascular disease risk factors in individuals with chronic spinal cord injury: a pilot study. Top Spinal Cord Inj Rehabil. 2014;20(2):127-36.
85. La Favor JD, Hollis BC, Mokshagundam SL, Olive JL. Serum hsCRP and visfatin are elevated and correlate to carotid arterial stiffness in spinal cord-injured subjects. Spinal Cord. 2011;49(9):961-6.
86. Edwards LA, Bugaresti JM, Buchholz AC. Visceral adipose tissue and the ratio of visceral to subcutaneous adipose tissue are greater in adults with than in those without spinal cord injury, despite matching waist circumferences. Am J Clin Nutr. 2008;87(3):600-7.
87. Iversen PO, Groot PD, Hjeltnes N, Andersen TO, Mowinckel MC, Sandset PM. Impaired circadian variations of haemostatic and fibrinolytic parameters in tetraplegia. Br J Haematol. 2002;119(4):1011-6.
88. Invernizzi M, Carda S, Rizzi M, Grana E, Squarzanti DF, Cisari C, Molinari C, Reno F. Evaluation of serum myostatin and sclerostin levels in chronic spinal cord injured patients. Spinal Cord. 2015;53(8):615-20.
89. Schreiber R, Paim LR, de Rossi G, Matos-Souza JR, Costa ESAA, Nogueira CD, Azevedo ER, Alonso KC, Palomino Z, Sposito AC, et al. Reduced Sympathetic Stimulus and Angiotensin 1-7 Are Related to Diastolic Dysfunction in Spinal Cord-Injured Subjects. J Neurotrauma. 2017;34(15):2323-8.
90. Bernardi M, Fedullo AL, Di Giacinto B, Squeo MR, Aiello P, Dante D, Romano S, Magaudda L, Peluso I, Palmery M, et al. Cardiovascular Risk Factors and Haematological Indexes of Inflammation in Paralympic Athletes with Different Motor Impairments. Oxidative Medicine and Cellular Longevity. 2019;2019 (no pagination).
